# Root developmental zonation is independent of cell wall pH

**DOI:** 10.1038/s41477-026-02339-z

**Published:** 2026-07-17

**Authors:** Pavel Krupař, Wiebke Haeger, Lorena Huffer, Daša Wernerová, Radek Vítek, Matouš Glanc, Thorsten Hamann, Matyáš Fendrych

**Affiliations:** 1 Institute of Experimental Botany of the Czech Academy of Sciences, Prague, Czech Republic; 2 Department of Experimental Plant Biology, Faculty of Science, Charles University, Prague, Czech Republic; 3 Institute for Biology, Faculty of Natural Sciences, Norwegian University of Science and Technology, Trondheim, Norway; 4 Department of Genetics and Breeding, Faculty of Agrobiology, Food and Natural Resources, Czech University of Life Sciences, Prague, Czech Republic

**Keywords:** Cell wall, Auxin

## Abstract

Plant development and morphogenesis rely on tight spatiotemporal regulation of cell growth, which in turn depends on the acidity of the cell wall^1,2^. According to the widely accepted acid growth theory, auxin promotes shoot cell expansion by acidifying the apoplast via activation of AHA proton pumps^3^. Nonetheless, how exactly auxin signalling and cell wall acidity feed into the developmental zonation of cell expansion in the roots remains unresolved. Here we show that while on the organ level apoplast acidification promotes root growth, the cellular surface pH and strain rate profiles are largely independent of each other. We introduce WALLΦ, a genetically encoded fluorescent sensor for non-invasive cell wall pH measurements. Using WALLΦ, we examined the cell wall pH gradients in *Arabidopsis* on the organ and cellular scale and found, contrary to the acid growth theory, a lack of a clear correlation of local growth rate with cell wall acidity. Globally, cell wall pH gates growth and allows for quick adaptations of growth rate and directionality.

## Abstract (web summary)

The authors developed a cell wall-anchored pH sensor and found that the cell elongation profile does not correlate with cell wall acidification in *Arabidopsis* roots. At the same time, cell wall pH changes allow for quick adaptations of growth rate and direction.

## Main

Plant morphogenesis relies on precise control of cell division and cell growth. In the root of the model plant *Arabidopsis thaliana*, cell division and cell elongation occur in distinct, well-defined zones along the longitudinal axis: the meristematic and elongation zone, respectively. This developmental zonation is regulated by the phytohormone auxin^4,5^.

At the cellular level, growth depends on the balance between the turgor pressure and the tensile strength of the cell wall (CW)^2,6^. In the classical ‘acid growth’ model, auxin activates plasma membrane H^+^-ATPases (AHAs) that acidify the apoplast, creating optimal conditions for EXPANSIN proteins, which in turn loosen the CW and induce growth in above-ground organs^1^.

The interplay between auxin signalling, CW pH and cell elongation in roots remains unclear. First, there are conflicting reports on the relationship between CW acidification and root growth^7–10^. Second, the role of auxin in the regulation of root CW pH is complex. The application of external auxin generally triggers root apoplast alkalinization and inhibits growth^9,11–14^. Auxin signalling mutants were shown to have both less acidic^13^ and more acidic apoplastic pH^9^. Moreover, the longitudinal cell elongation and pH profiles do not align with the acid growth model as cells grow most in the early elongation zone, which has a relatively high pH^9,11^. In this study, we aim to clarify the relationship between CW pH and root elongation. To achieve this, we developed a non-invasive tool for measuring CW pH in muro, and we reveal intriguing differences in CW pH on the organ and subcellular scale.

To dissect the effects of auxin signalling and apoplast acidity on cell elongation, it is crucial to relate them to the local cell expansion rate, rather than cell length^15^.

**Fig. 1:**
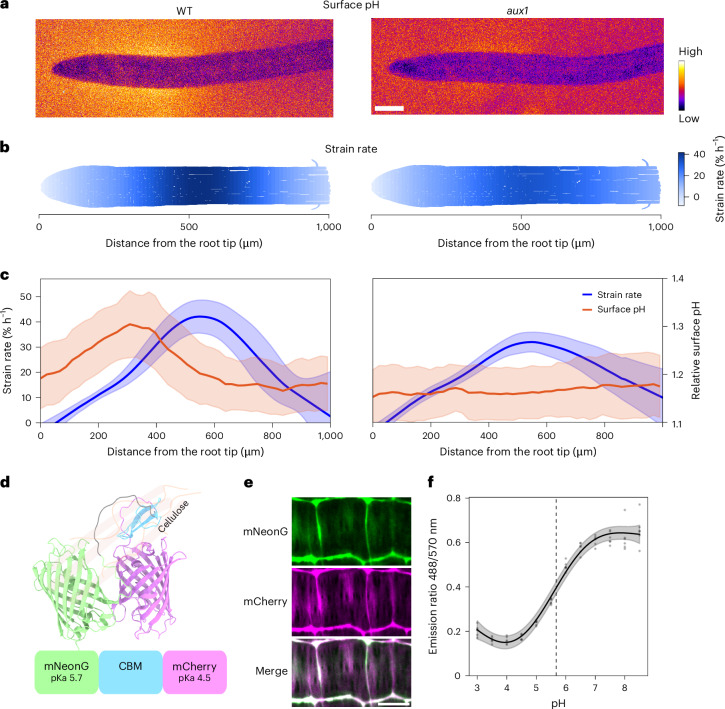
Root strain rate does not correlate with root surface pH profile. **a**, Representative ratiometric image of surface pH along the root longitudinal axis of WT and *aux1* mutant visualized by the pH-sensitive dye FS. The root colour does not reflect pH. Scale bar, 100 μm. **b**, A visualization of local strain rates in the roots of WT (left) and the *aux1* mutant (right). The map of strain rates was derived from analysis of brightfield images. Strain rate values were obtained as a derivation of velocity measures along the root longitudinal axis. **c**, The comparison of strain rate and surface pH profiles in WT (left) and *aux1* mutant (right) roots. For surface pH, *n* = 21–25, for strain rate, *n* = 19. The solid line represents the mean and the shaded area indicates the s.d. **d**, A schematic view and AlphaFold3 model of the WALLΦ CW pH sensor consisting of a CBM and two fluorescent proteins, mNeonGreen and mCherry. **e**, Root epidermal cell expressing WALLΦ showing a pattern resembling cellulose microfibrils. Scale bar, 10 μm. This experiment was repeated three times with similar results. **f**, In vitro calibration curve of CW-bound protein fraction isolated from WALLΦ expressing plants. Sample size, *n* = 7. The solid line is a Gaussian process regression; shading represents the 95% confidence interval. The dotted line indicates the estimated p*K*_a_ of 5.68. Panel **b** adapted from ref. ^51^ under a Creative Commons license CC-BY 3.0.

**Extended Data Fig. 1:**
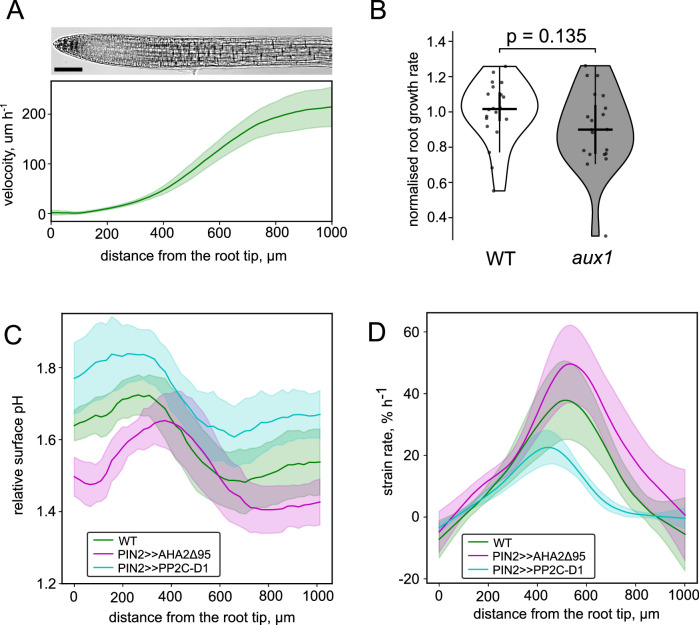
Manipulation of proton pump activity correlates with strain rate. **a**) Representative brightfield image and quantification of velocity along the root longitudinal axis of 5-day-old wild type plants. The values represent an average from 5 individual timepoints. Sample size: n = 19 roots. Scale bar = 100 μm. **b**) Normalized root growth rate of wild type (WT) and aux1 mutant plants. Values were normalized to the mean of the wild type control. Violin plots show the distribution of values per group. Horizontal lines represent the median, vertical bars show the interquartile range (IQR), and whiskers represent 1.5×IQR. **c**) Relative surface pH along the root longitudinal axis of lines with modified function of AHA2 - PIN2::AHA2delta95 and PIN2::PP2C-D1 visualized by FS dye. Sample size: n = 23-25 roots. **d**) Strain rate along the root longitudinal axis of lines with modified function of AHA2 - PIN2»AHA2delta95 and PIN2»PP2C-D1. Sample size: n = 8-16 roots.

To analyse the relationship of growth and root surface pH locally, we employed the strain rate, calculated as derivation of longitudinal velocity, reflecting the local rate of cell expansion. We combined particle image velocimetry (PIV) analysis^16^ (Extended Data Fig. 1a) with live imaging of a pH-sensitive dye: fluorescein-5-(and-6)-sulfonic acid, trisodium salt (FS)^9^. Notably, we observed no correlation between strain rate and root surface acidification along the longitudinal developmental axis; the zone with the highest strain rate exhibited higher pH, while the differentiation zone, where strain rate dropped to a minimum, was more acidic (Fig. 1a–c). This is in agreement with previous results^9,11,15^.

The domain with highest pH in the root transition zone depends on the auxin signalling pathway^9^. Mutants in the AUX1 auxin influx carrier have dramatically decreased auxin levels in root epidermis, and their surface pH profile is more acidic than wild type (WT)^9,11^. In contrast to the prediction of the acid growth theory, *aux1* exhibited a strain rate profile comparable to the WT. The mutant had a lower maximal strain rate compared to the WT, and the elongation zone was more spread along the longitudinal axis (Fig. 1c). The longer elongation zone in *aux1* probably compensates for the lower maximal strain rate, as the root elongation of *aux1* mutant was not different from WT (Extended Data Fig. 1b). These results demonstrate the lack of a straightforward correlation between root surface pH and cell elongation rates along the developmental axis of the root.

Does that mean the acid growth theory needs to be revised in the case of roots? To test the assumption of the acid growth theory, we used transgenic lines inducibly expressing a hyperactive plasma membrane proton pump AHA2 (PIN2»AHA2Δ95) and the negative regulator of AHAs, PP2C-D1 (PIN2»PP2C-D1), which were shown to affect root surface pH^9^. The manipulation of AHA activity did not alter the overall shape of the root surface pH profile, as shown previously^9^. Nevertheless, the hyperactive AHA2 acidified the root surface, and expression of AHA inhibitor led to surface alkalinization (Extended Data Fig. 1c). In agreement with this, the maximal strain rate and the size of the elongation zone increased with AHA2 activation and decreased with AHA inhibition (Extended Data Fig. 1d). Overall, despite the lack of spatial correlation between the local strain rate and surface pH, the AHA-mediated surface pH changes were sufficient to alter overall growth of the organ, in agreement with the acid growth theory.

The reason for the lack of correlation might be differences between the pH of the CW, directly implied in the control of cell expansion via the pH-dependent activity of EXPANSINs^17^ and the root surface pH. There have been reports suggesting a pH gradient exists within the apoplastic space, and pH inside the CW can be substantially different from that on the root surface^18,19^.

**Extended Data Fig. 2:**
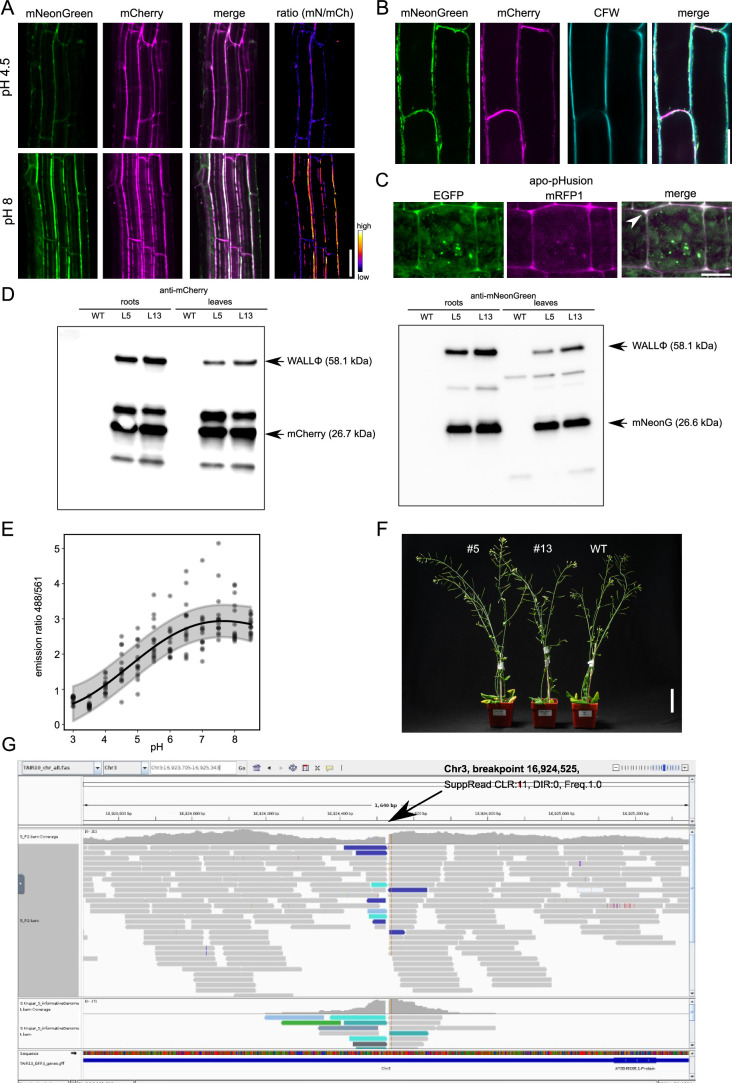
Characterization of WALLΦ pH sensor. **a**) Arabidopsis root expressing WALLΦ incubated in a calibration buffer of pH 4.5 and 8. The ratiometric image was obtained by segmentation of the original image using the cell wall mCherry signal to exclude cytoplasmic signal, and then by dividing mNeonGreen/mCherry signals. Scale = 50 μm. **b**) Colocalization of WALLΦ with cellulose stained by Calcofluor white (CFW) staining of root expressing WALLΦ. Scale = 20 μm. **c**) Representative image of a root epidermal cell expressing pH sensor ApopHusion. Arrow points to intercellular space. Scale = 10 μm. **d**) Western blot of protein fraction from WALLΦ roots and leaves, detected with anti-mCherry and anti-mNeonGreen antibodies. Wild type (WT) is used as a negative control. The antibody recognized intact WALLΦ protein and products of its degradation. **e**) in vivo calibration curve of 5-day-old seedlings expressing WALLΦ. Seedlings were incubated in a calibration buffer for 15 min and imaged. The solid line is a Gaussian-process regression; shading represents the 95% confidence interval. Sample size: n = 14. **f**) Phenotype of mature Arabidopsis plants expressing WALLΦ, two independent lines #5 and #13, compared to wild type (WT). Scale = 5 cm. **g**) Whole genome sequencing of WALLΦ line 5 detected an insert position on chromosome 3, breakpoint 16,924,525.

To determine CW pH directly at the site of EXPANSIN activity, we developed the wall-anchored pH indicator (WALLΦ), a genetically encoded ratiometric pH sensor targeted to cellulose microfibrils. We fused the carbohydrate-binding module (CBM) from the *Trichoderma reesei* CEL7a cellobiohydrolase^20^ (Extended Data Table 1) to mNeonGreen^21^ and mCherry^22^ fluorescent proteins, selected for their highly pH-dependent and practically pH-independent emission at physiological pH, respectively (analogously to the previously published apo-pHusion sensor^12^) (Fig. 1d and Extended Data Fig. 2a). We generated stable *Arabidopsis* lines expressing the WALLΦ reporter from the *Ubi4-2* promoter. Calcofluor white (CFW) staining^23^ confirmed that the WALLΦ protein colocalized with cellulose in vivo (Extended Data Fig. 2b) and we observed a visible pattern resembling cellulose microfibrils on the cell surface (Fig. 1e), which was not detectable in apo-pHusion lines (Extended Data Fig. 2c). Compared with the apo-pHusion, the presence of CBM targets WALLΦ to the CW, while apo-pHusion accumulated more in the intercellular spaces (Extended Data Fig. 2c and Supplementary Videos 1 and 2). The intact WALLΦ fusion protein was detected in roots and leaves (Extended Data Fig. 2d), with the root extracts containing relatively more intact protein than the leaf extract. In addition to the CW signal, we observed intracellular fluorescence, which was dominated by the mNeonGreen signal (Supplementary Videos 1 and 3). This signal originated presumably from the endoplasmic reticulum, where mNeonGreen was more visible due to its shorter maturation time and higher p*K*_a_ than mCherry, which strongly favours its fluorescence in the alkaline environment of the endoplasmic reticulum^24,25^. The intracellular signal of WALLΦ obscures the desired CW signal and therefore must be removed during the image analysis process (see below).

To analyse the WALLΦ pH response curve in vitro, we isolated the CW protein fraction^26,27^ and determined the mNeonGreen/mCherry emission ratios across pH values ranging from 3 to 8.5. Using the CW protein isolation protocol ensured that the vast majority of the signal originated from the CW-bound proteins, while most cytoplasmic proteins were washed out^26^. From the calibration curve, we estimated a p*K*_a_ of 5.68 (Fig. 1f), making the sensor suitable for rather acidic root apoplastic pH^19,28^. Further, we obtained the in vivo calibration curve by incubating plants in a growth medium adjusted to different pH values (Extended Data Fig. 2e). The values were measured from ratiometric images segmented on the CW signal and obtained in the root elongation/differentiation zone. Importantly, the transgenic plants showed no obvious developmental defects (Extended Data Fig. 2f) and whole-genome sequencing of line 5 revealed that the T-DNA insertion did not disrupt any known coding sequence (Extended Data Fig. 2g). In summary, we developed and characterized a novel CW-bound pH sensor suitable for in vivo CW pH estimations in real time.

**Fig. 2:**
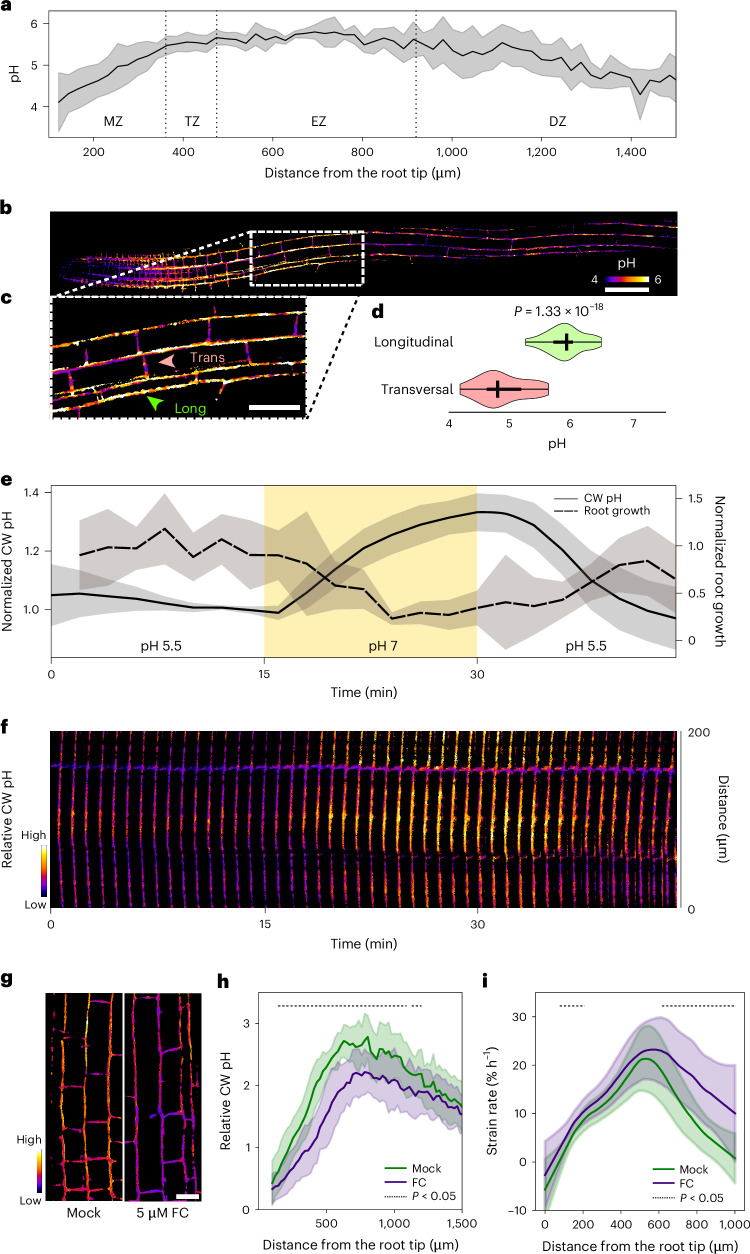
Root growth responds to apoplastic pH changes, despite a lack of correlation with the longitudinal CW pH profile. **a**,**b**, Quantification (**a**) and a representative image (**b**) of the root CW pH along the root longitudinal axis in the meristematic zone (MZ), transition zone (TZ), elongation zone (EZ) and differentiation zone (DZ). The units represent absolute pH values. The solid line represents the mean and the shaded area represents the s.d. Sample size in **a**, *n* = 5. Scale bar, 100 μm. **c**,**d**, An example image (**c**) and quantification (**d**) of longitudinal CWs (long), which are more alkaline than transversal walls (trans). CW pH of longitudinal (green arrow, long) and transversal CW segments (red arrow, trans). Scale bar, 50 μm. Sample size, *n* = 58 (longitudinal) and 47 (transversal) CW segments from 6 roots, *P* value according to two-sided Welch’s *t*-test. Violin plots show the distribution of values per group. Vertical lines represent the median, horizontal bars show the interquartile range (IQR) and whiskers represent 1.5× IQR. **e**, Relative CW pH and root growth rate over time react to external pH changes. Plants were mounted in a microfluidic chip and treated with a medium of pH 5.5 or 7. For the quantification, *n* = 8. Values were normalized on average of 5 timepoints before the first treatment. The solid line represents the mean and the shaded area represents the s.d. **f**, Kymograph of a representative ratiometric image showing a section of the root elongation zone. **g**,**h**, Representative ratiometric image (**g**) and quantification (**h**) of CW pH in the root elongation zone after 0 or 5 μM FC treatment. Sample size, *n* = 19–21; scale bar, 25 μm. **i**, The strain rate of seedlings expressing WALLΦ after 0 or 5 μM FC treatment. Sample size, *n* = 19–21. The solid line represents the mean and the shaded area indicates the s.d. In **h** and **i**, the statistical differences with *P* < 0.05 according to two-sided Welch’s *t*-test are indicated by a dotted line.

To investigate the relationship between root CW pH and cell elongation rate, we used WALLΦ to obtain a CW pH profile along the root longitudinal axis. First, to estimate absolute root CW pH, we imaged roots expressing WALLΦ growing vertically on unbuffered growth media. We converted the ratiometric images of CW segments on the epidermis/cortex boundary into a map of absolute pH values (Fig. 2a) using the *in vivo* calibration curve (Extended Data Fig. 2e). Nonetheless, converting the WALLΦ signal intensity ratio to absolute pH should be considered an estimate, as it might differ between experiments and based on the calibration used.

**Extended Data Fig. 3:**
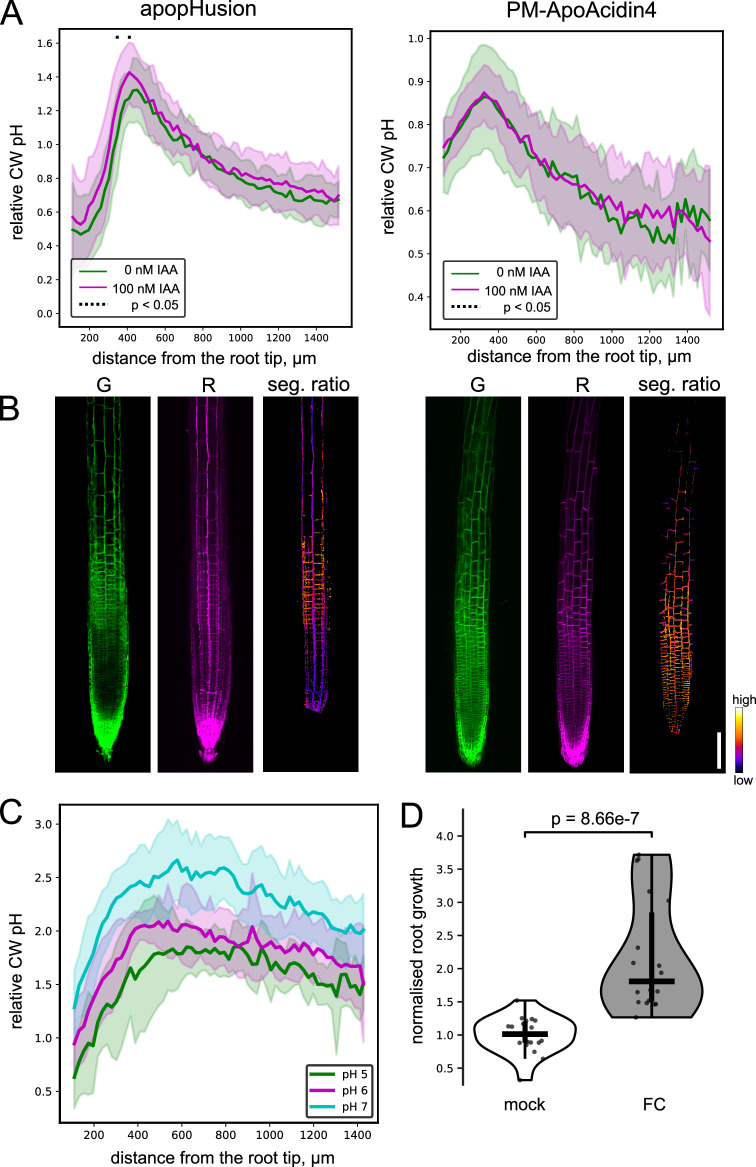
Longitudinal cell wall pH profile visualised by apopHusion and PM-ApoAcidin4. **a**) Relative apoplastic pH along the longitudinal root axis estimated by pH sensors ApopHusion and PM-ApoAcidin4 after 0 or 100 nM IAA treatment. Sample size: n = 14-17. **b**) Representative image of 5-day-old seedlings expressing apopHusion (left) and PM-ApoAcidin4 (right). Scale bar = 100 μm. **c**) Relative cell wall pH along the root longitudinal axis visualized by WALLΦ placed on media with indicated pH. n = 10-13. **d**) Normalized root growth rate of 5-day-old seedlings expressing WALLΦ after mock or 5 μM FC treatment. Values were normalized to the mean of the mock control. P-value according to Mann–Whitney U test, as the samples do not follow a normal distribution. Sample size: n = 18. Violin plots show the distribution of values per group. Horizontal lines represent the median, vertical bars show the interquartile range (IQR), and whiskers represent 1.5×IQR.

The root epidermis CW pH gradually increased from 4.5 in the meristem to 5.5 in the transition and early elongation zone before dropping back to 4.5 in the differentiation zone (Fig. 2b). In contrast to previously published studies describing the root elongation zone as the most acidic^13,29^, our data show that the CW of the early elongation zone has the highest pH among the root developmental zones (Fig. 2a,b). Different targeting of the pH sensors could possibly affect the sensor readouts^18,19^. We therefore analysed the previously published pH sensors: PM-ApoAcidin4, bound to the outer side of the plasma membrane^29^ and apo-pHusion, freely secreted into the apoplast^12^. Nonetheless, in our experimental conditions, both sensors showed a root apoplastic pH profile similar to the root surface pH profile, with the root transition zone having the highest pH and gradual acidification towards the root differentiation zone (Extended Data Fig. 3a,b).

When analysing the WALLΦ roots, we noticed conspicuous pH gradients within individual cells. The longitudinal CW segments consistently exhibited higher pH than the transversal ones (Fig. 2c,d). This was mostly apparent in the elongation zone, and a similar pattern is recognizable in roots expressing PM-ApoAcidin4 (Extended Data Fig. 3b). This is counterintuitive, given that the root elongates along its longitudinal axis. Overall, our observations of CW pH gradients on the cellular and organ level do not align with the paradigm that the local level of acidity of the wall can predict its elongation rate.

It was shown earlier that the manipulation of external pH changes root elongation rate^13,14^. To analyse the dynamics of the WALLΦ reaction and the corresponding root growth rate, we altered the external pH. Upon increasing the pH of the growth medium from 5.5 to 7, the CW pH in the elongation zone increased, while the root growth rate dropped (Fig. 2e,f). Upon subsequent re-acidification of the medium, CW pH and root growth rate gradually returned to their initial values. To understand how individual root zones react to the acidification of the external medium, we incubated seedlings expressing WALLΦ on solid medium with pH 5, 6, and 7. All developmental zones reacted equally to pH changes of the medium (Extended Data Fig. 3c). These experiments demonstrated that WALLΦ reports pH changes rapidly and reversibly. Further, we activated AHAs using the fungal toxin fusicoccin (FC). FC treatment triggered an overall CW acidification, but preserved the shape of the longitudinal pH profile with the highest pH in the transition and early elongation zone (Fig. 2g,h and Extended Data Table 2)^9^. Notably, the substantial increase in root growth upon FC treatment (Extended Data Fig. 3d) was due to a broader domain with high strain rate, rather than an increase in maximum strain rate values (Fig. 2i), resembling the strain profile of the *aux1* mutant.

Overall, these results demonstrate that the longitudinal CW pH profile is similar to that of the root surface. Further, while the manipulation of CW pH alters overall cell elongation, the correlation of CW acidity with elongation rate is not apparent neither on the developmental axis nor on the subcellular scale.

**Fig. 3:**
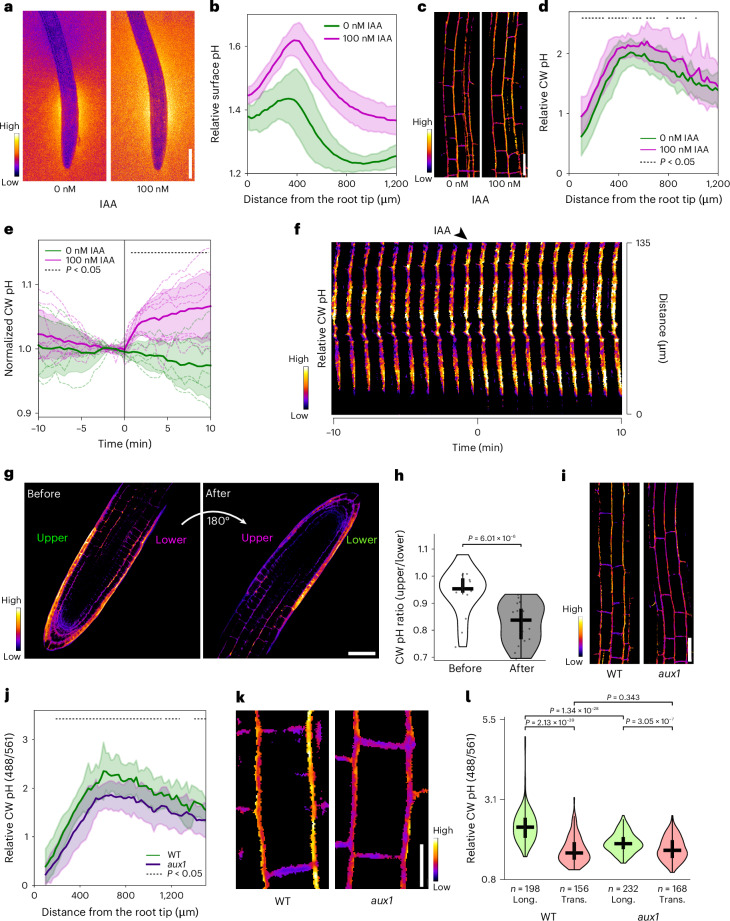
AUX1-mediated auxin influx is needed for the establishment of a normal root longitudinal CW pH profile. **a**,**b**, Representative image (**a**) and quantification (**b**) of relative root surface pH visualized by FS dye after treatment with 0 or 100 nM IAA. Sample size, *n* = 16–17. The solid line represents the mean, the shaded area represents the s.d. Scale bar, 200 μm. **c**,**d**, Representative image (**c**) and quantification (**d**) of CW pH along the root longitudinal axis visualized by WALLΦ after treatment with 0 or 100 nM IAA. Sample size, *n* = 21. *P* value based on two-sided Welch *t*-test indicated by the horizontal dotted line. Scale bar, 50 μm. **e**,**f**, Quantification (**e**) and kymograph (**f**) of pH of the root elongation zone visualized by WALLΦ after 0 or 100 nM IAA treatment in time. Plants were grown in a microfluidic chip. Treatment by IAA is visualized by a vertical line and an arrow. The solid line represents the mean, the dashed lines individual measurements and the shaded area represents the s.d. Sample size, *n* = 5–8. **g**,**h**, Representative image (**g**) and quantification (**h**) of relative CW pH in lateral roots of seedlings expressing WALLΦ before and after gravistimulation. Lateral roots were gravistimulated by rotating 180° for 30 min. Scale bar, 50 μm. Quantification of CW pH in the upper/lower side of the lateral root before and after gravistimulation is shown in **h**. Sample size, *n* = 16, *P* value according to a two-sided paired *t*-test. Shapiro–Wilk test on paired differences (before–after), *P* = 0.720. Violin plots show the distribution of values per group. Horizontal lines represent the median, vertical bars show the IQR and whiskers represent 1.5× IQR. **i**,**j**, Representative picture (**i**) and quantification (**j**) of relative CW pH along the root longitudinal axis of *aux1* mutant and WT visualized by WALLΦ. Sample size, *n* = 17–18. The solid line represents the mean and the shaded area represents the s.d. The *P* value based on two-sided Welch *t*-test is indicated by the horizontal dotted line. Scale bar, 50 μm. **k**,**l**, Representative image (**k**) and quantification (**l**) of relative CW pH of longitudinal (green) and transversal (red) CW segments in WT and *aux1* mutant visualized by WALLΦ. Violin plots show the distribution of values per group. Horizontal lines represent the median, vertical bars show the IQR and whiskers represent 1.5× IQR. A two-way ANOVA was used to assess the effects of genotype and CW orientation and their interaction. Pairwise comparisons were performed using two-sided Welch’s *t*-tests with Holm correction to account for unequal variances and multiple testing. Interaction, *P* = 1.13 × 10^−19^. Sample size, *n* = 156–232 CW segments from 15–18 roots. Scale bar, 20 μm.

**Extended Data Fig. 4:**
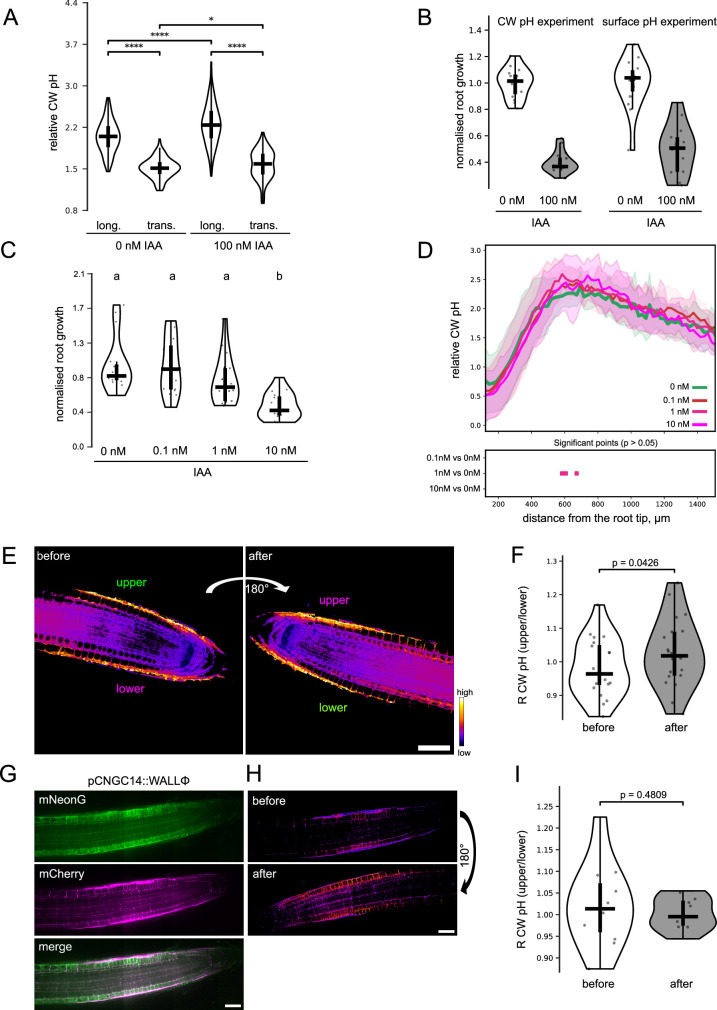
Effect of auxin on cell wall pH. **a**) Relative CW pH of individual root longitudinal or transversal CW segments after treatment with 0 nM or 100 nM IAA. Model assumptions were tested for normality of residues and for homogeneity of variance. Two-way ANOVA with heteroscedasticity-robust (HC3) standard errors. Pairwise comparisons were performed using Welch’s t-tests with Holm correction to account for unequal variances and multiple testing. Interaction: p = 7.38e-4. Sample size: 138–187 CW segments from 15-16 roots. Violin plots show the distribution of values per group. Horizontal lines represent the median, vertical bars show the interquartile range (IQR), and whiskers represent 1.5×IQR. Significance is indicated as follows: *p < 0.05, **p < 0.01, ***p < 0.001, ****p < 0.0001; ns, not significant. **b**) Normalized root growth rate of 5-day-old seedlings expressing WALLΦ after 0 nM or 100 nM IAA treatment. Surface pH experiment refers to the measurement of surface pH (see Fig. 3a,b), CW pH experiment refers to the measurement of cell wall pH (see Fig. 3c,d). Values were normalized to the mean of the mock control. **c**) Normalised root growth rate of 5-day-old seedling expressing WALLΦ after IAA treatment. Statistical analysis was performed using the non-parametric Kruskal–Wallis test followed by Dunn’s post hoc test with Holm correction for multiple comparisons. Groups sharing the same letter are not significantly different at p < 0.05. Values were normalized to the mean of the mock control. Sample size: n = 12-15. **d**) Relative CW pH profile of five-day-old seedlings expressing WALLΦ after IAA treatments. Each treatment was compared to the mock control using two-sided Welch’s t-tests. P-values were adjusted using the Benjamini–Hochberg false discovery rate procedure (FDR). Mean profiles are shown with shaded bands indicating SD, and the significance panel marks positions with FDR-adjusted p < 0.05. **e,f**) Relative cell wall pH visualized by WALLΦ in upper or lower segments of lateral root cap of a gravistimulated primary root. (**e**) a representative image and (F) quantification. n = 16, scale bar = 50 μm. **g**) Root tip expressing WALLΦ under pCNGC14 promoter. Scale bar = 50. H,I) Relative cell wall pH visualised by pCNGC14::WALLΦ in upper or lower segments of root epidermis before or after flipping by 180° (**h**) Representative image and (**i**) quantification. Scale bar = 50 μm.

Auxin induces rapid and extensive alkalinization of the root surface^9,11,14^. To assess whether this alkalinization is also reflected in the CW pH, we compared the reaction of the root surface pH measured by FS and CW pH estimated by WALLΦ upon external 100 nM IAA treatment. While the root surface exhibited a profound alkalinization (Fig. 3a,b), the reaction of the CW was mild, at the threshold of statistical significance (Fig. 3c,d). Notably, auxin treatment affected longitudinal CW segments stronger than transversal ones (Extended Data Fig. 4a) and the treatment was sufficient to strongly inhibit root elongation (Extended Data Fig. 4b). We also conducted this experiment with the previously published pH sensors PM-ApoAcidin4 and apo-pHusion. The reaction of both pH sensors to auxin was barely detectable. Alkalinization of the transition zone of apo-pHusion was on the threshold of statistical significance. (Extended Data Fig. 3a). To test whether low auxin concentrations promote root elongation, we estimated CW pH by WALLΦ in roots treated by 0.1 nM, 1 nM and 10 nM concentrations. While 0.1 nM and 1 nM concentrations had no significant effect on root elongation, 10 nM IAA inhibited root growth (Extended Data Fig. 4c). Also, lower auxin concentrations had no significant effect on CW pH, although a slight trend towards alkalinization was observed in the elongation zone (Extended Data Fig. 4d). To reveal the temporal dynamics of auxin-induced CW alkalinization, we imaged the WALLΦ sensor in a microfluidic chip. The CW alkalinization induced by 100 nM IAA was immediate (Fig. 3e,f), but relatively mild compared with previous studies^12,14^. This may be explained by WALLΦ localization in the CW matrix. As WALLΦ is bound to the cellulose microfibrils, proximity to CW structural components can significantly affect its pH readouts. Apoplastic space between cortex and epidermis is a site of strong buffering and will probably preserve its pH milieu than more distal apoplastic regions^18^. The existence of such pH gradients in the apoplast raises a question of how pH is regulated at the site of EXPANSIN action, at the cellulose microfibrils^17^.

While treatment with external 100 nM auxin elicited a CW pH increase, we aimed to analyse pH changes associated with natural auxin gradients that emerge during root gravitropic bending or in auxin transport mutants^30^. During gravitropic bending, auxin accumulates at the lower side of the root, where it inhibits growth^31,32^. The growth inhibition is accompanied by rapid changes in apoplastic pH in the root cap^33^, elongation zone^13^ and root surface^9,11^. We gravistimulated primary roots of 5-day-old seedlings expressing WALLΦ by rotating them by 90° for 15 min and then flipping them by 180°, as described in ref. ^34^, and imaged them before and after flipping. Contrary to the previous studies^9,11,33^, we did not detect a pH gradient in the CW between the lower and upper sides of the root (Extended Data Fig. 4e,f). We expressed the WALLΦ sensor from the pCNGC14, a weak promoter specific for the outer root tissues^9^, to overcome the strong lateral root cap signal of the ubiquitin promoter that might mask the CW pH gradients. The pCNGC14::WALLΦ lines showed a weak, tissue specific expression, nonetheless no CW pH gradient was detectable upon gravistimulation of the primary roots (Extended Data Fig. 4g–i). By contrast, after gravistimulation of young lateral roots, root tips exhibited profound acidification of their upper side (Fig. 3g,h) that coincides with the depletion of auxin^32^. Alkalinization of the lower side, where auxin accumulates, was not apparent; it is therefore possible that the growth gradient in the lateral root is driven by the expansion of the upper root side^32,35^. We conclude that the cuticle in lateral roots—a barrier that regulates the movement of water and ions^36^—creates a closed microenvironment, preventing proton extrusion to the root surface. The difference in pH changes between primary and lateral root can be explained by proton fluxes across the membrane, which may be restricted by the cuticle in lateral roots.

Finally, to better understand how the lack of auxin in root epidermis affects CW pH, we analysed the *aux1* mutant. Its CW pH profile was more acidic than that of WT roots, but preserves the normal pH zonation (Fig. 3i,j). Interestingly, while the surface pH profile of *aux1* lacks the domain with higher pH at the root transition zone (Fig. 1a,c), its CW pH keeps the longitudinal pH zonation comparable to WT, but is shifted towards more acidic values. This observation again points to differences in root surface and CW pH. We noticed that the longitudinal CW segments of *aux1* were more acidic than in the WT, while the pH of transversal CWs was comparable to WT (Fig. 3k,l). This suggests the role of auxin in specific alkalinization of longitudinal CW segments.

Genetically encoded fluorescent pH biosensors have become an indispensable tool for estimating pH in living organisms on the subcellular, cellular and organ scale^12^. WALLΦ ensures measurements directly in the CW matrix, but as well as its predecessors, it carries certain important limitations. The most problematic one is the intracellular signal of the sensor that originates from the endomembrane signal and the endoplasmic reticulum, where the sensor is maturing on its way towards the CW. This signal obscures the desired CW signal and complicates its pH estimation. While we reduced the noise signal by image analysis and segmentation of the CW signal, the contamination of the segmented images might cause inaccuracies in the measurements. Nevertheless, the CW pH profile that we observed fits the previously published reports^9,11,33^, and WALLΦ signal exhibits reactivity to pharmacological treatments that affect specifically the CW environment, confirming that WALLΦ visualizes real changes in CW pH rather than technical artefacts. Moreover, the lifetime of fluorophores excreted in the apoplastic space may also affect measurements. CW domains with intensive exocytosis might contain more of freshly secreted sensor, which might bias the sensor readout. This might apply to the pH difference between longitudinal and transversal CWs, nevertheless, the lack of this pH gradient in the *aux1* mutant strongly argues against this being a secretion artefact.

Taken together, we have clarified the relationships between auxin signalling, CW pH and root cell elongation using a novel CW pH sensor, WALLΦ, in combination with detailed cell strain rate profiling. We have demonstrated that CW pH changes are sufficient, but not necessary, to alter root cell elongation, and there is not a straightforward correlation between CW pH and cell elongation along the developmental axis of the root. While validating the classical acid growth model in the roots, these findings imply that the developmental zonation of the primary root depends on other mechanisms regulating cellular differentiation^37^.

## Methods

### Molecular cloning

To construct the WALLΦ pH indicator, we fused the ‘MMRARVPLLLLGILFLASLSASFA’ signal peptide, CBM from Cel7a^20^ (Extended Data Table 1) and mCherry in the following order:signal peptide, mNeonGreen^21^, CBM, mCherry (Addgene, pICSL50004^38^) under a parsley Ubi4-2 promoter (Addgene, pJOG020GB^38^). The construct was assembled with Ubq3 terminator into the pDGB3omega1 binary vector with a BASTA resistance cassette for plants using the GoldenBraid cloning system^39^. Coding sequences of mNeonGreen witch signal peptide, CBMs (Extended Data Table 1) were synthesized as DNA fragments (GeneArt Strings, Invitrogen). Coding sequences for CBM from EXPA7 (AT1G12560) and WAK1 (AT1G21250) sequences were cloned from genomic DNA using following primers: WAK1 forward-′GCGCCGTCTCGCTCGCCATGAAGGTGCAGGAGGGTTT′, WAK1 reverse-′GCGCCGTCTCGCTCACATTGAGAGACAAGTGCTGTTCCCAC′, EXPA7 forward-′GCGCCGTCTCGCTCGAATGGTGCCATGCCAAAGGAG′, EXPA7 reverse-′GCGCCGTCTCGCTCACGAACCACGGAAATTAGCGGTGCTCT′. The sequences include cloning restriction sites at their ends. To create plants expressing WALLΦ in root outer tissues, we cloned WALLΦ under the *CNGC14* promoter as described in ref. ^9^.

### Plant material and growth conditions

We used *A. thaliana*, ecotype Columbia (Col-0). To create two stable independent lines (5 and 13) carrying the WALLΦ construct. Plants were transformed using the floral dip method^40^. Unless specified otherwise, the experiments were performed with line 5. The following previously published lines were used: PIN2>>AHA2Δ95, PIN2>>PP2C-D1 (ref. ^9^), PM-ApoAcidin4 (ref. ^29^) and apo-pHusion^12^. WALLΦ was introduced into the *aux1-100* mutant (SALK_020355) by crossing. Seeds were sterilized by chlorine gas for 2 h and plated on half MS medium, 1% agar (Duchefa), 0.05% MES and 1% sucrose, pH 5.8 adjusted by KOH. Plates were stratified for 48 h at 4 °C and grown vertically in a growth chamber at 21 °C and 16 h of light per day.

### Chemical treatments

The following chemicals were used for treatments: IAA (10 mM stock in 96% ethanol, Sigma-Aldrich), 100 nM final concentration in unbuffered half MS, imaged immediately after plating seedling on treatment medium; FC (1 mM stock in 96% ethanol, Sigma-Aldrich), 5 µM final concentration, imaged after 15 min after plating seedlings on treatment medium; estradiol (20 mM stock in DMSO, Sigma) 5 µM final concentration in half MS, imaged after 4 h treatment; CFW, 0.01% final concentration in liquid half MS medium, imaged after 30 min; fluorescein dextran 10,000 MW, anionic (D1821, Thermo Fisher) 10 mg ml^−1^ stock in H_2_O, final concentration 29 μg ml^−1^; FS (F1130, Thermo Fisher), 50 mM stock in H_2_O, final concentration 50 μM in unbuffered half MS medium pH 5.6 adjusted with KOH or HCl.

### Imaging and microfluidics

The imaging was performed using Zeiss Axio Observer 7 confocal microscope with a Yokogawa CSU-W1-T2 spinning disk unit equipped with objectives Zeiss Plan-Apochromat ×20/0.8 for CW pH measurements, Zeiss Plan-Apochromat ×10/0.45 for surface pH and strain rate measurements, LD LCI Plan-Apochromat 40×/1.2 with glycerol immersion for gravitropic experiments. The sample was illuminated by a LED lamp (PIV), 488 nm (WALLΦ, apo-Phusion, PM-ApoAcidin4) and 561 nm (WALLΦ, apo-pHusion, PM-ApoAcidin4) lasers.

The signal was captured by a PRIME-95B Back-Illuminated sCMOS camera (Photometrics) or Orca Flash 4.0 V3 Hamamatsu camera for PIV.

For gravitropic experiments of the lateral roots, 10-day-old seedlings expressing WALLΦ grown vertically were mounted to a microscopic chamber covered by a layer of solid half MS medium, pH 5.8. The seedlings were left to recover for 30 min and imaged. For primary roots, 5-day-old seedlings expressing WALLΦ grown vertically were mounted to a microscopic chamber covered by a layer of solid half MS medium, pH 5.8 and the chamber was rotated by 90° and after 15 min imaged as before stimulation. Then the chamber was flipped by 180° and left 30 min for further stimulation.

For CFW staining and Supplementary Video 3, we used a Zeiss LSM 900 laser scanning microscope (Axio Observer 7, inverted) equipped with a high-resolution Airyscan2 detector, and LD LCI Plan-Apo 40×/1.2 objective. For high-resolution images of cellulose microfibrils, we used Mirava Polyscope superresolution system with STED laser equipped with objective 60×/1.2, UPLSAPO60XW with water immersion. Five-day-old plants were mounted to a microscope slide covered by cover slip and incubated in half MS medium, pH 7, buffered by MES. For mCherry, we used a deactivation 775 nm STED laser. For timelapse videos of growing plants expressing WALLΦ and apo-pHusion (Supplementary Videos 1 and 2), we used the PlantScope Xero system with a Yokogawa CSU-W1 spinning disk equipped with Zeiss Plan-Apochromat 20×/0.8 objective. Plants were mounted to a microscopic chamber on a slice of solid half MS medium without sucrose. For excitation of mNeonGreen and eGFP we used a 488 nm laser, and for mCherry and mRFP1, we used a 561 nm laser. For image acquisition, we used the following software: VisiView (Visitron) for Zeiss Axio Observer 7, SlideBook (3i Intelligent Imaging Innovations) for PlantScope, LiGHTBOX (Abberior) for Mirava Polyscope and ZEN Blue (Zeiss) for LSM 900.

For microfluidics experiments, 5-day-old plants were mounted to a microfluidics chip described in ref. ^41^. The system consisted of two channels, each for one treatment. The control medium contained fluorescein dextran and media exchange was indicated by its intensity. Flow rate was set to 3 μl min^−1^. For pH and growth experiments, we used liquid half MS medium, 1% sucrose, buffered with 0.05% MES with pH adjusted to 5.5 (by HCl) or 7 (by KOH). For IAA treatment, we used liquid unbuffered half MS medium,1% sucrose with a final concentration of 100 nM IAA in the medium, or an equivalent volume of ethanol.

### Image analysis

Image processing was performed using ImageJ Fiji software^42^. For strain rate measurements, images were registered as the root tip remained in fixed position and rotated to the same orientation. To measure longitudinal velocity, we used PIVlab software v.2.63^16^ running on MATLAB R2022a (MathWorks, v.9.12). The velocity was measured using the FFT window deformation algorithm in two passes with an interrogation area 64 px for pass 1 and 40 px for pass 2. The step was set to 50 px for pass 1 and 20 px for pass 2. Velocity data were shown as average from ten individual timepoints within a 1 min time period. Longitudinal velocity values were converted to strain rate using the Savitzky–Golay filter (Scipy Signal Python library) with a window length of 21 and polynomial 2. For microfluidics experiments, the image was registered so the root elongation zone stayed in fixed position and the fluorescence ratio over time measured in a rectangular selection at the root elongation zone. The values were normalized on average of the first five timepoints. Root growth was measured from brightfield images by manual tracking of the root tip over time. Root increment was measured as a root tip displacement over one timepoint in Fiji.

### pH measurements

For surface pH measurements, 5-day-old seedlings were moved on unbuffered half MS medium, 1% (w/v) sucrose, pH 5.6, 0.8% agar (Duchefa) and 50 µM FS. The surface pH along the root longitudinal axis was estimated as the fluorescence ratio using a Python script Along the Root, described in ref. ^9^.

For CW pH measurements, the roots were excited by 488 nm (mNeonGreen) and 561 nm (mCherry) lasers with 20× magnification. Multiple images were captured along the root longitudinal axis and stitched using the Fiji Pairwise stitching plugin^43^. Images were segmented using the 561 nm channel to exclude the cytoplasmic signal visible in the 488 nm channel only. As the signal at the root cap was significantly stronger and incomparable with other tissues, the area of the root cap (first 100 μm) was cropped out for measuring root longitudinal pH profiles. From segmented images, we created a ratio image by dividing the 488 nm/561 nm channels. The root longitudinal pH profile was measured as a segmented line along the root. The fluorescence values along the longitudinal axis were averaged in bins of size 40 pixels. In quantifications, each value represents one bin. This protocol was used also for obtaining root longitudinal pH profiles with other published pH sensors. Alternatively, for gravitropic experiments, the signal was measured only in the lateral root cap from the ratiometric image. For measurements of CW pH of longitudinal and transversal CW segments, we used a segmented line selection covering the CW segment in the root elongation zone. The average value was measured for each CW segment.

### Protein extraction and western blot

For in vitro WALLΦ sensor calibration, we used a CW protein isolation protocol as described by ref. ^27^, which was originally adapted from ref. ^26^. The published protocol was followed with specific modifications: above-ground organs of *Arabidopsis* expressing WALLΦ were harvested at the rosette developmental stage and immediately frozen in liquid nitrogen and stored at −80 °C. For 48 g of frozen plant material, 1,500 ml of extraction buffer was used. Protein extraction proceeded according to the original protocol; however, only the protein fraction extracted with 0.2 M CaCl_2_ was collected and subsequently lyophilized. Before fluorescence measurements, the lyophilized protein was resuspended in ultrapure water.

For western blot, the roots and leaves from 7-day-old seedlings were separated, frozen in liquid nitrogen and homogenized. The sample was incubated for 30 min at room temperature with a Tris-extraction buffer (30 mM TrisHCl, 10% glycerol, 150 mM NaCl, 10 mM EDTA and 1 mM DTT, 2% cOmplete protease inhibitor (Roche)) and centrifuged at 14,000 rpm (rcf of 16,653*g*) for 15 min at 4 °C. Protein concentration was adjusted to 4 μg of protein per sample. The samples were denatured by boiling with a 6× SDS loading buffer and separated by PAGE on 4–20% TGX StainFree gel (Bio-Rad). The separated proteins were blotted to a nitrocellulose membrane and incubated with primary polyclonal antibodies against mCherry (AbCam, ab167453, diluted 1:8,000), mNeonGreen (Proteintech, 29523-1-AP, diluted 1:10,000) and secondary antibody goat anti-Rabbit IgG (H + L), (Invitrogen, 65-6120, diluted 1:10,000). The membranes were incubated with antibodies in 3% dried non-fat milk at room temperature for 1 h. The signal was detected using the ECL kit (Fastgene) and imaged on chemiluminescence imaging system (Bio-Rad).

### pH sensor calibration in vivo and in vitro

To calibrate the WALLΦ pH sensor in vivo, we prepared a calibration buffer containing 10 mM MES, MOPS and trisodium citrate; the pH was adjusted in range 3−8.5 by KOH or HCl. Five-day-old seedlings were mounted to microscopy glass and incubated in the buffer for 15 min. The fluorescence ratio was measured in the root elongation zone (see ‘pH measurements’ section).

For in vitro calibration, we used a protein fraction isolated from the CW (see ‘Protein extraction and western blot’ section). The isolated protein was resuspended in ultrapure water and diluted 1:10 in the calibration buffer. The fluorescence was measured on a Cytation 5 fluorescence plate reader (Agilent). For mNeonGreen the excitation/emission wavelengths were 488 nm/517 nm and for mCherry 570 nm/610 nm.

Calibration curves relating fluorescence intensity to pH were fitted using Gaussian process regression implemented in scikit-learn (v.1.6.1^44^). A composite kernel was used, consisting of a radial basis function kernel and a WhiteKernel to account for noise. The Gaussian process was trained on pooled fluorescence values from 7 (in vitro) or 14 (in vivo) replicates for each pH calibration point. The Gaussian process model was used to generate a smooth, non-parametric estimate of the fluorescence response as a function of pH, with 95% confidence intervals. The p*K*_a_ was defined as the pH at which the Gaussian process-predicted fluorescence reached half-maximal intensity. To convert fluorescence ratio values to pH, we used a Python script (NumPy library) to interpolate the values based on the Gaussian process calibration curve. All fluorescence values outside of the calibration range (corresponding to pH 6.5 or higher) were excluded. The pH values were reshaped to a two-dimensional image, where each pixel holds a pH value and saved as a TIFF file.

### Whole-genome sequencing

DNA was isolated from leaf segments of 5-day-old plants using a modified CTAB extraction protocol^45^. Libraries were prepared from ~100 ng of DNA using the KAPA EvoPlus V2 kit. We used five amplification cycles. Libraries were sequenced on NovaSeq X Plus. Reads were trimmed using the fastp program^46^ and analysed using TDNAscan^47^ to identify T-DNA insertion and mapped to TAIR10 reference genome^48^.

### Statistics and graphics

The presented data consist of a mix of biological and technical replicates. All experiments except microfluidics and conversion to absolute pH values were conducted in three biological replicates. The *n* of the samples are indicated in the figure legends. The *P* values are indicated in the figures or in the Extended Data Table 2. The microfluidics experiments were conducted from at least three independent chips, using seedlings plated at least two different days. The conversion of relative to absolute pH values served only to provide approximation and was measured from two biological replicates. The main conclusions are based on the consistent relative profiles obtained across all experiments. For protein modelling, Alphafold3 running on an alphafold server^49^ was used, running in default settings of five replicates. The best scoring model (ranked by pLDDT score) was used. All statistical analyses and plots were performed using Python 3.11 executed in Google Colaboratory (Google LLC) using following libraries, scikit-learn (1.6.1), matplotlib (3.10.0), pandas (2.2.2), seaborn (0.13.2), numpy (2.0.2) and scipy (1.16.1). ChatGPT (OpenAI) was used for support in code development and troubleshooting.

### Reporting summary

Further information on research design is available in the Nature Portfolio Reporting Summary linked to this article.

## Supplementary information

Reporting Summary

Peer Review File

Supplementary Video 1 A time-lapse video of *Arabidopsis* root expressing WALLΦ (mNeonGreen, mCherry, merge and segmented ratio channels shown from left to right).

Supplementary Video 2 A time-lapse video of *Arabidopsis* root expressing apo-pHusion (eGFP, mRFP1, merge and segmented ratio channels shown from left to right).

Supplementary Video 3 Video of *Arabidopsis* root cells expressing WALLΦ (mNeonGreen and mCherry).

## Data Availability

Source data used to generate the figures are available via Zenodo at 10.5281/zenodo.19812494 (ref. ^50^).

## Extended data

**Extended Data Table 1:** Carbohydrate binding modules (CBM) used for cloning of a pH sensor

CBM	Organism of origin	Binding	Localization	Stable trans.	UniProt ID	Sequence
Cel7a	*Trichoderma reesei*	cellulose	CW	YES	G0RVK1	SHYGQCGGIGYSGPTVCASGTTCQVLNPYYSQCLGS
CBM3a	*Clostridium thermocellum*	cellulose	CW	YES	Q06851	SGNLKVEFYNSNPSDTTNSINPQFKVTNTGSSAIDLSKLTLRYYYTVDGQKDQTFWCDHAAIIGSNGSYNGITSNVKGTFVKMSSSTNNADTYLEISFTGGTLEPGAHVQIQGRFAKNDWSNYTQSNDYSFKSASQFVEWDQVTAYLNGVLVWGKEP
CBM2b	*Cellulomonas fimi*	xylan	CW	YES	P54865	MGGGGGGGGGTGSCSVSAVRGEEWADRFNVTYSVSGSSSWVVTLGLNGGQSVQSSWNAALTGSSGTVTARPNGSGNSFGVTFYKNGSSATPGATC
CFICEX	*Cellulomonas fimi*	cellulose, xylan	CW	NO	P07986	SGPAGCQVLWGVNQWNTGFTANVTVKNTSSAPVDGWTLTFSFPSGQQVTQAWSSTVTQSGSAVTVRNAPWNGSIPAGGTAQFGFNGSHTGTNAAPTAFSLNGTPCTVG
WAK1	*Arabidopsis thaliana*	unknown	cytoplasmic	NO	Q39191	MKVQEGLFLVAIFFSLACTQLVKGQHQPGENCQNKCGNITIEYPFGISSGCYYPGNESFSITCKEDRPHVLSDIEVANFNHSGQLQVLLNRSSTCYDEQGKKTEEDSSFTLENLSLSANNKLTAVGCNALSLLDTFGMQNYSTACLSLCDSPPEADGECNGRGCCRVDVSAPLDSYTFETTSGRIKHMTSFHDFSPCTYAFLVEDDKFNFSSTEDLLNLRNVMRFPVLLDWSVGNQTCEQVGSTSICGGNSTCL
Expa7	*Arabidopsis thaliana*	unknown	vacuolar	NO	Q9LN94	MVPCQRSGGMRFQFQGNSYWLLIFVMNVGGAGDIKSMAVKGSRTNWISMSHNWGASYQAFSSLYGQSLSFRVTSYTTGETIYAWNVAPANWSGGKTYKSTANFR

**Extended Data Table 2:** Statistical data

Distance/Time	p_value_welch	significant_p_lt_0.05
0	0.186916	FALSE
13	0.160856	FALSE
26	0.133949	FALSE
39	0.106841	FALSE
52	0.080526	FALSE
65	0.056362	FALSE
78	0.03592	TRUE
91	0.020553	TRUE
104	0.010715	TRUE
117	0.005573	TRUE
130	0.003563	TRUE
143	0.003844	TRUE
156	0.006176	TRUE
169	0.012412	TRUE
182	0.021728	TRUE
195	0.031967	TRUE
208	0.045057	TRUE
221	0.058075	FALSE
234	0.06872	FALSE
247	0.067309	FALSE
260	0.05826	FALSE
273	0.058765	FALSE
286	0.057943	FALSE
299	0.060804	FALSE
312	0.057182	FALSE
325	0.060425	FALSE
338	0.055236	FALSE
351	0.058833	FALSE
364	0.060589	FALSE
377	0.057051	FALSE
390	0.072364	FALSE
403	0.085973	FALSE
416	0.108424	FALSE
429	0.139965	FALSE
442	0.171615	FALSE
455	0.231759	FALSE
468	0.318525	FALSE
481	0.398889	FALSE
494	0.461558	FALSE
507	0.477218	FALSE
520	0.456964	FALSE
533	0.419443	FALSE
546	0.39137	FALSE
559	0.33434	FALSE
572	0.269761	FALSE
585	0.195418	FALSE
598	0.135718	FALSE
611	0.081637	FALSE
624	0.049996	TRUE
637	0.02771	TRUE
650	0.014174	TRUE
663	0.009141	TRUE
676	0.005987	TRUE
689	0.003832	TRUE
702	0.0025	TRUE
715	0.00144	TRUE
728	0.000827	TRUE
741	0.000531	TRUE
754	0.000291	TRUE
767	0.000199	TRUE
780	0.000121	TRUE
793	8.54E-05	TRUE
806	7.37E-05	TRUE
819	6.44E-05	TRUE
832	5.34E-05	TRUE
845	5.09E-05	TRUE
858	4.53E-05	TRUE
871	4.01E-05	TRUE
884	4.6E-05	TRUE
897	5.77E-05	TRUE
910	7.83E-05	TRUE
923	0.000113	TRUE
936	0.000171	TRUE
949	0.000266	TRUE
962	0.000419	TRUE
975	0.00066	TRUE
988	0.001033	TRUE
1001	0.001589	TRUE

## References

[1] Arsuffi, G. & Braybrook, S. A. Acid growth: an ongoing trip. *J. Exp. Bot.* **69**, 137–146 (2018). 29211894 10.1093/jxb/erx390

[2] Braidwood, L., Breuer, C. & Sugimoto, K. My body is a cage: mechanisms and modulation of plant cell growth. *New Phytol.* **201**, 388–402 (2014). 24033322 10.1111/nph.12473

[3] Takahashi, K., Hayashi, K.-I. & Kinoshita, T. Auxin activates the plasma membrane H^+^-ATPase by phosphorylation during hypocotyl elongation in *Arabidopsis*. *Plant Physiol.* **159**, 632–641 (2012). 22492846 10.1104/pp.112.196428 PMC3375930

[4] Berleth, T. & Jürgens, G. The role of the *monopteros* gene in organising the basal body region of the *Arabidopsis* embryo. *Development* **118**, 575–587 (1993).

[5] Roychoudhry, S. & Kepinski, S. Auxin in root development. *Cold Spring Harb. Perspect. Biol.* **14**, a039933 (2022). 34312248 10.1101/cshperspect.a039933 PMC9121899

[6] Lintilhac, P. M. The problem of morphogenesis: unscripted biophysical control systems in plants. *Protoplasma* **251**, 25–36 (2014). 23846861 10.1007/s00709-013-0522-y PMC3893470

[7] Diaz-Ardila, H. N. & Hardtke, C. S. Extracellular pH, cell length and cell differentiation do not firmly correlate across *Arabidopsis* root tissues. *Plant Cell Physiol.*10.1093/pcp/pcaf031 (2025). 40126926 10.1093/pcp/pcaf031 PMC12290282

[8] Pacheco-Villalobos, D. et al. The effects of high steady state auxin levels on root cell elongation in brachypodium. *Plant Cell* **28**, 1009–1024 (2016). 27169463 10.1105/tpc.15.01057 PMC4904674

[9] Serre, N. B. C. et al. The AUX1–AFB1–CNGC14 module establishes a longitudinal root surface pH profile. *eLife* **12**, e85193 (2023). 37449525 10.7554/eLife.85193 PMC10414970

[10] Peters, W. S. & Felle, H. H. The correlation of profiles of surface pH and elongation growth in maize roots. *Plant Physiol.* **121**, 905–912 (1999). 10557239 10.1104/pp.121.3.905 PMC59453

[11] Monshausen, G. B., Miller, N. D., Murphy, A. S. & Gilroy, S. Dynamics of auxin-dependent Ca2^+^ and pH signaling in root growth revealed by integrating high-resolution imaging with automated computer vision-based analysis. *Plant J.* **65**, 309–318 (2011). 21223394 10.1111/j.1365-313X.2010.04423.x

[12] Gjetting, K. S. K., Ytting, C. K., Schulz, A. & Fuglsang, A. T. Live imaging of intra- and extracellular pH in plants using pHusion, a novel genetically encoded biosensor. *J. Exp. Bot.* **63**, 3207–3218 (2012). 22407646 10.1093/jxb/ers040 PMC3350929

[13] Barbez, E., Dünser, K., Gaidora, A., Lendl, T. & Busch, W. Auxin steers root cell expansion via apoplastic pH regulation in *Arabidopsis thaliana*. *Proc. Natl Acad. Sci. USA* **114**, E4884–E4893 (2017). 28559333 10.1073/pnas.1613499114 PMC5474774

[14] Li, L. et al. Cell surface and intracellular auxin signalling for H^+^ fluxes in root growth. *Nature* **599**, 273–277 (2021). 34707283 10.1038/s41586-021-04037-6 PMC7612300

[15] Beemster, G. T. & Baskin, T. I. Analysis of cell division and elongation underlying the developmental acceleration of root growth in *Arabidopsis thaliana*. *Plant Physiol.* **116**, 1515–1526 (1998). 9536070 10.1104/pp.116.4.1515 PMC35060

[16] Thielicke, W. & Sonntag, R. Particle image velocimetry for MATLAB: accuracy and enhanced algorithms in PIVlab. *J. Open Res. Softw.* **9**, 12 (2021).

[17] Wang, T. et al. Sensitivity-enhanced solid-state NMR detection of expansin’s target in plant cell walls. *Proc. Natl Acad. Sci. USA* **110**, 16444–16449 (2013). 24065828 10.1073/pnas.1316290110 PMC3799313

[18] Felle, H. The apoplastic pH of the *Zea mays* root cortex as measured with pH-sensitive microelectrodes: aspects of regulation. *J. Exp. Bot.* **49**, 987–995 (1998).

[19] Martinière, A. et al. Uncovering pH at both sides of the root plasma membrane interface using noninvasive imaging. *Proc. Natl Acad. Sci. USA* **115**, 6488–6493 (2018). 29866831 10.1073/pnas.1721769115 PMC6016826

[20] Guo, J. & Catchmark, J. M. Binding specificity and thermodynamics of cellulose-binding modules from *Trichoderma reesei* Cel7A and Cel6A. *Biomacromolecules* **14**, 1268–1277 (2013). 23506559 10.1021/bm300810t

[21] Shaner, N. C. et al. A bright monomeric green fluorescent protein derived from *Branchiostoma lanceolatum*. *Nat. Methods* **10**, 407–409 (2013). 23524392 10.1038/nmeth.2413 PMC3811051

[22] Shaner, N. C. et al. Improved monomeric red, orange and yellow fluorescent proteins derived from *Discosoma* sp. red fluorescent protein. *Nat. Biotechnol.* **22**, 1567–1572 (2004). 15558047 10.1038/nbt1037

[23] Hughes, J. & McCully, M. E. The use of an optical brightener in the study of plant structure. *Stain Technol.* **50**, 319–329 (1975). 54956 10.3109/10520297509117082

[24] Shen, J. et al. Organelle pH in the *Arabidopsis* endomembrane system. *Mol. Plant* **6**, 1419–1437 (2013). 23702593 10.1093/mp/sst079

[25] Martinière, A. et al. In vivo intracellular pH measurements in tobacco and *Arabidopsis* reveal an unexpected pH gradient in the endomembrane system. *Plant Cell* **25**, 4028–4043 (2013). 24104564 10.1105/tpc.113.116897 PMC3877828

[26] Feiz, L., Irshad, M., Pont-Lezica, R. F., Canut, H. & Jamet, E. Evaluation of cell wall preparations for proteomics: a new procedure for purifying cell walls from *Arabidopsis* hypocotyls. *Plant Methods* **2**, 10 (2006). 16729891 10.1186/1746-4811-2-10 PMC1524762

[27] Haeger, W. et al. New players in the interaction between beetle polygalacturonases and plant polygalacturonase-inhibiting proteins: Insights from proteomics and gene expression analyses. *Front. Plant Sci.* **12**, 660430 (2021). 34149758 10.3389/fpls.2021.660430 PMC8213348

[28] Staal, M. et al. Apoplastic alkalinization is instrumental for the inhibition of cell elongation in the *Arabidopsis* root by the ethylene precursor 1-aminocyclopropane-1-carboxylic acid. *Plant Physiol.* **155**, 2049–2055 (2011). 21282405 10.1104/pp.110.168476 PMC3091085

[29] Moreau, H., Gaillard, I. & Paris, N. Genetically encoded fluorescent sensors adapted to acidic pH highlight subdomains within the plant cell apoplast. *J. Exp. Bot.* **73**, 6744–6757 (2022). 35604912 10.1093/jxb/erac210

[30] Swarup, R. et al. Localization of the auxin permease AUX1 suggests two functionally distinct hormone transport pathways operate in the *Arabidopsis* root apex. *Genes Dev.* **15**, 2648–2653 (2001). 11641271 10.1101/gad.210501 PMC312818

[31] Friml, J., Wiśniewska, J., Benková, E., Mendgen, K. & Palme, K. Lateral relocation of auxin efflux regulator PIN3 mediates tropism in *Arabidopsis*. *Nature* **415**, 806–809 (2002). 11845211 10.1038/415806a

[32] Band, L. R. et al. Root gravitropism is regulated by a transient lateral auxin gradient controlled by a tipping-point mechanism. *Proc. Natl Acad. Sci. USA* **109**, 4668–4673 (2012). 22393022 10.1073/pnas.1201498109 PMC3311388

[33] Fasano, J. M. et al. Changes in root cap pH are required for the gravity response of the *Arabidopsis* root. *Plant Cell* **13**, 907–921 (2001). 11283344 10.1105/tpc.13.4.907 PMC135544

[34] Kulich, I., Schmid, J., Teplova, A., Qi, L. & Friml, J. Rapid translocation of NGR proteins driving polarization of PIN-activating D6 protein kinase during root gravitropism. *Elife* **12**, RP91523 (2024). 38441122 10.7554/eLife.91523 PMC10942638

[35] Fendrych, M. et al. Rapid and reversible root growth inhibition by TIR1 auxin signalling. *Nat Plants* **4**, 453–459 (2018). 29942048 10.1038/s41477-018-0190-1 PMC6104345

[36] Berhin, A. et al. The root cap cuticle: a cell wall structure for seedling establishment and lateral root formation. *Cell* **176**, 1367–1378.e8 (2019). 30773319 10.1016/j.cell.2019.01.005

[37] Nolan, T. M. et al. Brassinosteroid gene regulatory networks at cellular resolution in the *Arabidopsis* root. *Science* **379**, eadf4721 (2023). 36996230 10.1126/science.adf4721 PMC10119888

[38] Gantner, J. et al. Peripheral infrastructure vectors and an extended set of plant parts for the modular cloning system. *PLoS ONE* **13**, e0197185 (2018). 29847550 10.1371/journal.pone.0197185 PMC5976141

[39] Sarrion-Perdigones, A. et al. GoldenBraid 2.0: a comprehensive DNA assembly framework for plant synthetic biology. *Plant Physiol.* **162**, 1618–1631 (2013). 23669743 10.1104/pp.113.217661 PMC3707536

[40] Clough, S. J. & Bent, A. F. Floral dip: a simplified method for *Agrobacterium*-mediated transformation of *Arabidopsis thaliana*. *Plant J.* **16**, 735–743 (1998). 10069079 10.1046/j.1365-313x.1998.00343.x

[41] Serre, N. B. C. et al. AFB1 controls rapid auxin signalling through membrane depolarization in *Arabidopsis thaliana* root. *Nat. Plants* **7**, 1229–1238 (2021). 34282287 10.1038/s41477-021-00969-z PMC7611683

[42] Schindelin, J. et al. Fiji: an open-source platform for biological-image analysis. *Nat. Methods* **9**, 676–682 (2012). 22743772 10.1038/nmeth.2019 PMC3855844

[43] Preibisch, S., Saalfeld, S. & Tomancak, P. Globally optimal stitching of tiled 3D microscopic image acquisitions. *Bioinformatics* **25**, 1463–1465 (2009). 19346324 10.1093/bioinformatics/btp184 PMC2682522

[44] Pedregosa, F. et al. Scikit-learn: machine learning in Python. *J. Mach. Learn.* **12**, 2825−2830 (2012).

[45] Doyle, J. J. & Doyle, J. L. Rapid DNA isolation procedure small quantities fresh leaf tissue. *Phytochem. Bull.* **19**, 11–15 (1987).

[46] Chen, S., Zhou, Y., Chen, Y. & Gu, J. fastp: an ultra-fast all-in-one FASTQ preprocessor. *Bioinformatics* **34**, i884–i890 (2018). 30423086 10.1093/bioinformatics/bty560 PMC6129281

[47] Sun, L. et al. TDNAscan: a software to identify complete and truncated T-DNA insertions. *Front. Genet.* **10**, 685 (2019). 31428129 10.3389/fgene.2019.00685 PMC6690219

[48] Lamesch, P. et al. The *Arabidopsis* Information Resource (TAIR): improved gene annotation and new tools. *Nucleic Acids Res.* **40**, D1202–D1210 (2012). 22140109 10.1093/nar/gkr1090 PMC3245047

[49] Abramson, J. et al. Accurate structure prediction of biomolecular interactions with AlphaFold 3. *Nature* **630**, 493–500 (2024). 38718835 10.1038/s41586-024-07487-w PMC11168924

[50] Krupar Pavel, F. M. Source data for the publication: root developmental zonation is independent of cell wall pH. *Zenodo*10.5281/zenodo.19812493 (2026). 10.1038/s41477-026-02339-z PMC13384898 42469378

[51] Bouché, F. *Arabidopsis* - roots: longitudinal section. *Figshare*10.6084/m9.figshare.4688809 (2017).

