# Supplementary material for: Root developmental zonation is independent of cell wall pH
Source: Nat Plants. 2026 Jul 17;12(7):1334–43. doi: 10.1038/s41477-026-02339-z (PMC13384898; doi:10.1038/s41477-026-02339-z)
Supplement: Supplementary file 1 — Reporting Summary [file 41477_2026_2339_MOESM1_ESM.pdf]

nature portfolio

Corresponding author(s): Matyáš Fendrych

Last updated by author(s): Mar 26, 2026

Reporting Summary

Nature Portfolio wishes to improve the reproducibility of the work that we publish. This form provides structure for consistency and transparency in reporting. For further information on Nature Portfolio policies, see our Editorial Policies and the Editorial Policy Checklist.

Statistics

For all statistical analyses, confirm that the following items are present in the figure legend, table legend, main text, or Methods section.

n/a	Confirmed	
☐	☒	The exact sample size (*n*) for each experimental group/condition, given as a discrete number and unit of measurement
☐	☒	A statement on whether measurements were taken from distinct samples or whether the same sample was measured repeatedly
☐	☒	The statistical test(s) used AND whether they are one- or two-sided<br>*Only common tests should be described solely by name; describe more complex techniques in the Methods section.*
☒	☐	A description of all covariates tested
☐	☒	A description of any assumptions or corrections, such as tests of normality and adjustment for multiple comparisons
☐	☒	A full description of the statistical parameters including central tendency (e.g. means) or other basic estimates (e.g. regression coefficient) AND variation (e.g. standard deviation) or associated estimates of uncertainty (e.g. confidence intervals)
☐	☒	For null hypothesis testing, the test statistic (e.g. *F*, *t*, *r*) with confidence intervals, effect sizes, degrees of freedom and *P* value noted<br>*Give P values as exact values whenever suitable.*
☒	☐	For Bayesian analysis, information on the choice of priors and Markov chain Monte Carlo settings
☒	☐	For hierarchical and complex designs, identification of the appropriate level for tests and full reporting of outcomes
☒	☐	Estimates of effect sizes (e.g. Cohen's *d*, Pearson's *r*), indicating how they were calculated

Our web collection on statistics for biologists contains articles on many of the points above.

Software and code

Policy information about availability of computer code

Data collection	VisiView (Visitron), LiGHTBOX (Abberior), ZEN Blue (Zeiss).
Data analysis	PIVlab (Thielicke and Sonntag 2021, v. 2.63) running on MATLAB R2022a (The MathWorks Inc., version 9.12), ImageJ Fiji v1.54 (Schindelin et al., 2012). Python 3.11 executed in Google Colaboratory (Google LLC) using following libraries, scikit-learn (1.6.1), matplotlib (3.10.0), pandas (2.2.2), seaborn (0.13.2), numpy (2.0.2), scipy (1.16.1).

For manuscripts utilizing custom algorithms or software that are central to the research but not yet described in published literature, software must be made available to editors and reviewers. We strongly encourage code deposition in a community repository (e.g. GitHub). See the Nature Portfolio guidelines for submitting code & software for further information.

Data

Policy information about availability of data

All manuscripts must include a data availability statement. This statement should provide the following information, where applicable:

- Accession codes, unique identifiers, or web links for publicly available datasets
- A description of any restrictions on data availability
- For clinical datasets or third party data, please ensure that the statement adheres to our policy

The WALLΦ lines will be deposited to the NASC database, and the raw data used to generate the images will be deposited to the Zenodo database before the manuscript will be published.

Research involving human participants, their data, or biological material

Policy information about studies with human participants or human data. See also policy information about sex, gender (identity/presentation), and sexual orientation and race, ethnicity and racism.

Reporting on sex and gender	*Use the terms sex (biological attribute) and gender (shaped by social and cultural circumstances) carefully in order to avoid confusing both terms. Indicate if findings apply to only one sex or gender; describe whether sex and gender were considered in study design; whether sex and/or gender was determined based on self-reporting or assigned and methods used. Provide in the source data disaggregated sex and gender data, where this information has been collected, and if consent has been obtained for sharing of individual-level data; provide overall numbers in this Reporting Summary. Please state if this information has not been collected. Report sex- and gender-based analyses where performed, justify reasons for lack of sex- and gender-based analysis.*
Reporting on race, ethnicity, or other socially relevant groupings	*Please specify the socially constructed or socially relevant categorization variable(s) used in your manuscript and explain why they were used. Please note that such variables should not be used as proxies for other socially constructed/relevant variables (for example, race or ethnicity should not be used as a proxy for socioeconomic status). Provide clear definitions of the relevant terms used, how they were provided (by the participants/respondents, the researchers, or third parties), and the method(s) used to classify people into the different categories (e.g. self-report, census or administrative data, social media data, etc.) Please provide details about how you controlled for confounding variables in your analyses.*
Population characteristics	*Describe the covariate-relevant population characteristics of the human research participants (e.g. age, genotypic information, past and current diagnosis and treatment categories). If you filled out the behavioural & social sciences study design questions and have nothing to add here, write "See above."*
Recruitment	*Describe how participants were recruited. Outline any potential self-selection bias or other biases that may be present and how these are likely to impact results.*
Ethics oversight	*Identify the organization(s) that approved the study protocol.*

Note that full information on the approval of the study protocol must also be provided in the manuscript.

Field-specific reporting

Please select the one below that is the best fit for your research. If you are not sure, read the appropriate sections before making your selection.

☒ Life sciences ☐ Behavioural & social sciences ☐ Ecological, evolutionary & environmental sciences

For a reference copy of the document with all sections, see nature.com/documents/nr-reporting-summary-flat.pdf

Life sciences study design

All studies must disclose on these points even when the disclosure is negative.

Sample size	The sample size was determined based on a pilot experiment, from which the variance and effect size for the primary outcome were estimated. The n of used samples are indicated in the figure legends.
Data exclusions	No data were excluded from the analysis.
Replication	All experiments except microfluidics and conversion to absolute pH values were conducted in three biological replicates. The microfluidics experiments were conducted from at least three independent chips, using seedlings plated at least two different days. The conversion of relative to absolute pH values served only to provide approximation and was measured from two biological replicates. The main conclusions are based on the consistent relative pH profiles obtained across all experiments.
Randomization	Plants were randomly assigned to treatment and control groups, and were grown on at least two independent plates to avoid positional bias.
Blinding	Blinding was not relevant for this study.

Reporting for specific materials, systems and methods

We require information from authors about some types of materials, experimental systems and methods used in many studies. Here, indicate whether each material, system or method listed is relevant to your study. If you are not sure if a list item applies to your research, read the appropriate section before selecting a response.

Materials & experimental systems

n/a	Involved in the study	
[ ]	[x]	Antibodies
[x]	[ ]	Eukaryotic cell lines
[x]	[ ]	Palaeontology and archaeology
[x]	[ ]	Animals and other organisms
[x]	[ ]	Clinical data
[x]	[ ]	Dual use research of concern
[ ]	[x]	Plants

Methods

n/a	Involved in the study	
[x]	[ ]	ChIP-seq
[x]	[ ]	Flow cytometry
[x]	[ ]	MRI-based neuroimaging

Antibodies

Antibodies used

polyclonal antibodies against mCherry (AbCam, ab167453), mNeonGreen (Proteintech, 29523-1-AP), secondary antibody goat anti-Rabbit IgG (H+L), (Invitrogen, 65-6120)

Validation

mCherry https://www.abcam.com/en-us/products/publications?productId=ab167453, mNeonGreen https://www.ptglab.com/products/mNeonGreen-Antibody-29523-1-AP.htm?srsltid=AfmBOorAgTvdSJSY1DNiuEKRpGgBlPp0PWmyaK_wOBoIvMRgYCSYTP7d#publications

Dual use research of concern

Policy information about dual use research of concern

Hazards

Could the accidental, deliberate or reckless misuse of agents or technologies generated in the work, or the application of information presented in the manuscript, pose a threat to:

No	Yes	
[ ]	[ ]	Public health
[ ]	[ ]	National security
[ ]	[ ]	Crops and/or livestock
[ ]	[ ]	Ecosystems
[ ]	[ ]	Any other significant area

Experiments of concern

Does the work involve any of these experiments of concern:

No	Yes	
[ ]	[ ]	Demonstrate how to render a vaccine ineffective
[ ]	[ ]	Confer resistance to therapeutically useful antibiotics or antiviral agents
[ ]	[ ]	Enhance the virulence of a pathogen or render a nonpathogen virulent
[ ]	[ ]	Increase transmissibility of a pathogen
[ ]	[ ]	Alter the host range of a pathogen
[ ]	[ ]	Enable evasion of diagnostic/detection modalities
[ ]	[ ]	Enable the weaponization of a biological agent or toxin
[ ]	[ ]	Any other potentially harmful combination of experiments and agents

Plants

Seed stocks	Arabidopsis thaliana, ecotype Columbia (Col-0), PIN2::AHA2delta95, PIN2::PP2C-D1 (Serre Wernerová), PM-ApoAcidin4 (Moreau, Gaillard, and Paris 2022), ApopHusion (Gjetting et al. 2012)
Novel plant genotypes	To construct the WALLΦ pH indicator, we fused a signal peptide, carbohydrate-binding module (CBM) from Cel7a (Supp.table1, Guo and Catchmark 2013) and mCherry in the following order: SP , mNeonGreen (Shaner et al. 2013) , CBM, mCherry (Addgene, pICSL50004, (Gantner et al. 2018) under a parsley Ubi 4-2 promoter (Addgene, pJOG020GB, (Gantner et al. 2018). The construct was assembled with Ubq3 terminator into the pDGB3omega1 binary vector with a BASTA resistance cassette for plants. Two stable independent lines (#5 and 13) carrying the WALLΦ construct were created using the floral dip method. WALLΦ was introduced into the aux1-100 mutant (SALK_020355) by crossing. To create plants expressing WALLΦ in root outer tissues, we cloned WALLΦ under the CNGC14 promoter (Serre,Wernerová 2023)
Authentication	Line 5 carrying WALLΦ was analyzed by whole genome sequencing, proving one T-DNA insertion in correct frequency and orientation.
