# Supplementary material for: Root developmental zonation is independent of cell wall pH
Source: Nat Plants. 2026 Jul 17;12(7):1334–43. doi: 10.1038/s41477-026-02339-z (PMC13384898; doi:10.1038/s41477-026-02339-z)
Supplement: Supplementary file 2 — Peer Review File [file 41477_2026_2339_MOESM2_ESM.pdf]

nature portfolio

Peer Review File

Root developmental zonation is independent of cell wall pH

Corresponding Author: Dr Matyas Fendrych

Version 0:

Reviewer comments:

Reviewer #1

(Comments for the Author)
In this manuscript, the authors measured the pH value in the cell walls of root tips using a novel fluorescent indicator. They also investigated the effect of auxin on apoplastic pH in root tips to evaluate the applicability of the classic "acid growth" theory. The findings contribute to our understanding of pH dynamics in the apoplast of various root tip cells and the relationship between apoplastic pH and root growth rate. The results are both novel and interesting. However, there are still some points that need to be addressed or clarified.

Major points:
1. The relationship between apoplastic pH and cell growth rate needs clarification. It appears that root cells in the elongation zone, which experience a higher strain rate, possess a higher cell wall pH. This observation suggests a negative correlation between cell wall pH and cell elongation rate. However, the cell elongation rate is higher in acidic conditions (pH 5.5) compared to neutral conditions (pH 7.0); cell wall acidification induced by FC promotes cell elongation; and IAA-induced cell wall alkalinization, indicating a potential positive correlation between cell wall acidification and cell elongation. These findings seem contradictory, making it difficult to draw a definitive conclusion. In my opinion, it is inappropriate to assert that "root elongation rate is independent of cell wall pH," as cell wall pH does indeed affect root growth.
2. The authors selected a concentration of 100 nM IAA to treat the roots, as this level appeared to inhibit root growth (as shown in Figure S4A). Root cells are more sensitive than those in other organs, and auxin promotes growth at low concentrations while inhibiting it at high concentrations. Therefore, it is important to investigate the effects of lower concentrations of IAA.
3.The authors observed an asymmetric distribution of cell wall pH at the tips of main roots and young lateral roots influenced by gravity; however, the results from the two experiments seemed inconsistent. The reason for such a discrepancy should be addressed in detail.

Minor points:
1. In Fig. 3E, it appears that the addition of 0 nM IAA leads to a decrease in pH. What could explain this observation?
2. In the legend of Fig. 2, line 265, the authors reference "In G and G." However, there is no statistical data presented in Fig. 2G.
3. In the Materials and Methods section, lines 292-314, the references are cited in a format that differs from that used in the main text.
4. In lines 326-327, the notation for "H2O" should have the "2" in subscript.
5. In the legend for Fig. S4, line 45, it mentions "Fig. 4AB" and "Fig. 4CD." However, I cannot find Fig. 4; should it be "Fig. S4"? If so, note that there is no figure D included in Fig. S4.
6. In Fig. S4A, it is difficult to understand how the root growth rates of "cell wall" and "surface" are measured. Could you clarify this data?

Reviewer #2

(Comments for the Author)
Using WALLΦ, a novel genetically encoded sensor the authors show that although apoplastic acidification promotes root growth at the organ level, local cell wall pH does not correlate with cellular expansion rates. They suggest that instead of directly instructing local growth, cell wall acidity acts as a global permissive factor that enables rapid adjustment of growth rate and direction. The relationship between auxin, cell wall pH and growth rate is central for understanding plant growth and morphogenesis and has received increasing attention in the past years. However, results using different methods to measure cell surface and apoplastic pH so far have not been conclusive and the field clearly is need of better tools. The chosen approach seems highly promising but several points need to be addressed before this ms becomes ready to go:

- Please describe how strain rate relates to growth velocity. This might be clear to the expert but not to the average

reader/reviewer.
- What is the evidence that WallPhi actually binds cellulose? Based on the images provided the signal is much more patchy than the CF signal.
- What makes it different from apoPhusion?
- Could the difference between lateral and horizontal wall be an imaging artifact based on different birefringence/material anisotropy?
- Call me old-fashioned but alkaline is reserved to pH>7, all measurements here show that the cell wall is more or less acidic.

Decision Letter:

22nd December 2025

Dear Dr Fendrych,

Your Letter, "Root developmental zonation is independent of cell wall pH" has now been seen by two referees. You will see from their comments below that while they find your work of interest, some important points are raised. We are interested in the possibility of publishing your study in Nature Plants, but would like to consider your response to these concerns in the form of a revised manuscript before we make a final decision on publication.

As you can read below, both reviewers think that the findings are interesting and novel, however, some concerns are raised which should be addressed. In our opinion the main points are:

1. Reviewer #1 states that the relationship between apoplastic pH and cell growth rate needs further clarification. The discrepancy between the distribution of cell wall pH at tips of main roots and lateral roots should be explained. Finally, reviewer #1 thinks that lower auxin concentrations should be used for root treatment.
2. Reviewer #2 thinks that evidence is required that WALLΦ binds cellulose, and it should be explained how WALLΦ differs from apo-pHusion. Furthermore, it should be explained how strain rate relates to growth velocity.

All referees also raise some other points that should be addressed. As usual, please do not hesitate to get in touch if you would like to discuss these issues further.

We therefore invite you to revise your manuscript taking into account all reviewer and editor comments. Please highlight all changes in the manuscript text file, preferably in Microsoft Word format.

We are committed to providing a fair and constructive peer-review process. Do not hesitate to contact us if there are specific requests from the reviewers that you believe are technically impossible or unlikely to yield a meaningful outcome.

When revising your manuscript please:

* Include a "Response to referees" document detailing, point-by-point, how you addressed each referee comment. If no action was taken to address a point, you must provide a compelling argument. This response will be sent back to the referees along with the revised manuscript.

* If you have not done so already please begin to revise your manuscript so that it conforms to our Letter format instructions at http://www.nature.com/nplants/info/final-submission. Refer also to any guidelines provided in this letter.

* Include a revised version of any required reporting checklist. It will be available to referees (and, potentially, statisticians) to aid in their evaluation if the manuscript goes back for peer review. A revised checklist is essential for re-review of the paper.

* Pay close attention to our href="https://www.nature.com/nature-portfolio/editorial-policies/image-integrity">Digital Image Integrity Guidelines. and to the following points. Please ensure:
-- that unprocessed scans are clearly labelled and match the gels and western blots presented in figures.
-- that control panels for gels and western blots are appropriately described as loading on sample processing controls
-- that all images in the paper are checked for duplication of panels and for splicing of gel lanes.
-- that you retain unprocessed data and metadata files after publication, ideally archiving data in perpetuity. These may be requested during the peer review and production process or after publication if any issues arise.

EXTENDED DATA FIGURES

When re-submitting your manuscript, please ensure that any supplementary figures and tables that are crucial to the manuscript's conclusions are converted into Extended Data figures and tables to increase visibility of these data. Extended Data figures and tables are online-only (present in the online PDF and full-text HTML versions of the paper), peer-reviewed display items that provide essential background to the article but are not included in the main article due to space constraints. A maximum of ten Extended Data display items (figures and tables) is permitted.

Nature Plants strongly supports public availability of data and are therefore keen that the data used in your paper is placed in an appropriate public data repository. Alternatively, if this is not possible, you may present the data as Extended Data or Supplementary Information. If data can only be shared on request, please explain why in your Data Availability Statement, and also in the correspondence with your editor. Please note that for some data types, deposition in a public repository is

mandatory.

Please use the link below to submit your revised manuscript and related files:

Link Redacted

Note: This URL links to your confidential home page and associated information about manuscripts you may have submitted, or that you are reviewing for us. If you wish to forward this email to co-authors, please delete the link to your homepage.

We hope to receive your revised manuscript within two to three months, however, this is not a strict deadline. If you cannot send it within this time, please let us know. We will be happy to consider your revision so long as nothing similar has been accepted for publication at Nature Plants or published elsewhere.

We look forward to seeing the revised manuscript and thank you for the opportunity to review your work.

Sincerely,

Nature Plants is committed to improving transparency in authorship. As part of our efforts in this direction, we are now requesting that all authors identified as 'corresponding author' on published papers create and link their Open Researcher and Contributor Identifier (ORCID) with their account on the Manuscript Tracking System (MTS), prior to acceptance. This applies to primary research papers only. ORCID helps the scientific community achieve unambiguous attribution of all scholarly contributions. You can create and link your ORCID from the home page of the MTS by clicking on 'Modify my Springer Nature account'. For more information please visit please visit www.springernature.com/orcid.

Please do not hesitate to contact me if you have any questions or would like to discuss these revisions further.

****

Reviewers' Comments:

Reviewer #1 (Comments for the Author):

In this manuscript, the authors measured the pH value in the cell walls of root tips using a novel fluorescent indicator. They also investigated the effect of auxin on apoplastic pH in root tips to evaluate the applicability of the classic “acid growth” theory. The findings contribute to our understanding of pH dynamics in the apoplast of various root tip cells and the relationship between apoplastic pH and root growth rate. The results are both novel and interesting. However, there are still some points that need to be addressed or clarified.

Major points:
1. The relationship between apoplastic pH and cell growth rate needs clarification. It appears that root cells in the elongation zone, which experience a higher strain rate, possess a higher cell wall pH. This observation suggests a negative correlation between cell wall pH and cell elongation rate. However, the cell elongation rate is higher in acidic conditions (pH 5.5) compared to neutral conditions (pH 7.0); cell wall acidification induced by FC promotes cell elongation; and IAA-induced cell wall alkalinization, indicating a potential positive correlation between cell wall acidification and cell elongation. These findings seem contradictory, making it difficult to draw a definitive conclusion. In my opinion, it is inappropriate to assert that "root elongation rate is independent of cell wall pH," as cell wall pH does indeed affect root growth.
2. The authors selected a concentration of 100 nM IAA to treat the roots, as this level appeared to inhibit root growth (as shown in Figure S4A). Root cells are more sensitive than those in other organs, and auxin promotes growth at low concentrations while inhibiting it at high concentrations. Therefore, it is important to investigate the effects of lower concentrations of IAA.
3.The authors observed an asymmetric distribution of cell wall pH at the tips of main roots and young lateral roots influenced by gravity; however, the results from the two experiments seemed inconsistent. The reason for such a discrepancy should be addressed in detail.

Minor points:
1. In Fig. 3E, it appears that the addition of 0 nM IAA leads to a decrease in pH. What could explain this observation?
2. In the legend of Fig. 2, line 265, the authors reference “In G and G.” However, there is no statistical data presented in Fig. 2G.
3. In the Materials and Methods section, lines 292-314, the references are cited in a format that differs from that used in the main text.
4. In lines 326-327, the notation for “H2O” should have the “2” in subscript.
5. In the legend for Fig. S4, line 45, it mentions “Fig. 4AB” and “Fig. 4CD.” However, I cannot find Fig. 4; should it be “Fig. S4”? If so, note that there is no figure D included in Fig. S4.
6. In Fig. S4A, it is difficult to understand how the root growth rates of “cell wall” and “surface” are measured. Could you clarify this data?

Reviewer #2 (Comments for the Author):

Using WALLΦ, a novel genetically encoded sensor the authors show that although apoplastic acidification promotes root growth at the organ level, local cell wall pH does not correlate with cellular expansion rates. They suggest that instead of directly instructing local growth, cell wall acidity acts as a global permissive factor that enables rapid adjustment of growth rate and direction. The relationship between auxin, cell wall pH and growth rate is central for understanding plant growth and morphogenesis and has received increasing attention in the past years. However, results using different methods to measure cell surface and apoplastic pH so far have not been conclusive and the field clearly is need of better tools. The chosen approach seems highly promising but several points need to be addressed before this ms becomes ready to go:

- Please describe how strain rate relates to growth velocity. This might be clear to the expert but not to the average reader/reviewer.
- What is the evidence that WallPhi actually binds cellulose? Based on the images provided the signal is much more patchy than the CF signal.
- What makes it different from apoPhusion?
- Could the difference between lateral and horizontal wall be an imaging artifact based on different birefringence/material anisotropy?
- Call me old-fashioned but alkaline is reserved to pH>7, all measurements here show that the cell wall is more or less acidic.

********************END********************

Version 1:

Reviewer comments:

Reviewer #1

(Comments for the Author)
The authors had revised the manuscript carefully. My original comments had been responded properly.

Reviewer #2

(Comments for the Author)
All my comments have been addressed and the ms has been further improved. From my perspective, the ms is ready for publication!

Decision Letter:

Our ref: NPLANTS-251121401A

21st April 2026

Dear Dr. Fendrych,

Thank you for submitting your revised manuscript "Root developmental zonation is independent of cell wall pH" (NPLANTS-251121401A). It has now been seen by the original referees and their comments are below. The reviewers find that the paper has improved in revision, and therefore we'll be happy in principle to publish it in Nature Plants.

If the current version of your manuscript is in a PDF format, please email us a copy of the file in an editable format (Microsoft Word or LaTex)-- we can not proceed with PDFs at this stage.

We are now performing detailed checks on your paper and will send you a checklist detailing our editorial and formatting requirements in about a week. Please do not upload the final materials and make any revisions until you receive this additional information from us.

Thank you again for your interest in Nature Plants Please do not hesitate to contact me if you have any questions.

Sincerely,

Reviewer #1 (Comments for the Author):

The authors had revised the manuscript carefully. My original comments had been responded properly.

Reviewer #2 (Comments for the Author):

All my comments have been addressed and the ms has been further improved. From my perspective, the ms is ready for publication!

Version 2:

Decision Letter:

8th June 2026

Dear Dr Fendrych,

We are pleased to inform you that your Letter entitled "Root developmental zonation is independent of cell wall pH", has now been accepted for publication in Nature Plants.

Over the next few weeks, your paper will be copyedited to ensure that it conforms to Nature Plants style. We look particularly carefully at the titles of all papers to ensure that they are relatively brief and understandable.

Once your paper is typeset, you will receive an email with a link to choose the appropriate publishing options for your paper and our Author Services team will be in touch regarding any additional information that may be required.

After the grant of rights is completed, you will receive a link to your electronic proof via email with a request to make any corrections within 48 hours. If, when you receive your proof, you cannot meet this deadline, please inform us at rjsproduction@springernature.com immediately.

You will not receive your proofs until the publishing agreement has been received through our system.

You may wish to make your media relations office aware of your accepted publication, in case they consider it appropriate to organize some internal or external publicity. Once your paper has been scheduled you will receive an email confirming the publication details. This is normally 3-4 working days in advance of publication. If you need additional notice of the date and time of publication, please let the production team know when you receive the proof of your article to ensure there is sufficient time to coordinate. Further information on our embargo policies can be found here: https://www.nature.com/authors/policies/embargo.html

Due to the importance of these deadlines, we ask you please us know now whether you will be difficult to contact over the next month. If this is the case, we ask you provide us with the contact information (email, phone and fax) of someone who will be able to check the proofs on your behalf, and who will be available to address any last-minute problems.

Acceptance of your manuscript is conditional on all authors' agreement with our publication policies (see http://www.nature.com/authors/policies/index.html). In particular your manuscript must not be published elsewhere.

If you have any questions about our publishing options, costs, Open Access requirements, or our legal forms, please contact ASJournals@springernature.com

Authors may need to take specific actions to achieve compliance with funder and institutional open access mandates. If your research is supported by a funder that requires immediate open access (e.g. according to Plan S principles or the NIH public access policy) then you should select the gold OA route, and we will direct you to the compliant route where possible. Because authors warrant under our subscription licensing terms that they haven’t committed to licensing any version of their article under a licence inconsistent with the terms of our agreement – including the applicable embargo period – publication under the subscription model isn’t suitable for authors whose funders require no embargo.

An online order form for reprints of your paper is available at https://www.nature.com/reprints/author-reprints.html. All co-authors, authors' institutions and authors' funding agencies can order reprints using the form appropriate to their geographical region.

We welcome the submission of potential cover material (including a short caption of around 40 words) related to your manuscript; suggestions should be sent to Nature Plants as electronic files (the image should be 300 dpi at 210 x 297 mm in either TIFF or JPEG format). Please note that such pictures should be selected more for their aesthetic appeal than for their scientific content, and that colour images work better than black and white or grayscale images. Please include a written

description of your image and how it was created along with your image files. Please do not try to design a cover with the Nature Plants logo etc., and please do not submit composites of images related to your work. I am sure you will understand that we cannot make any promise as to whether any of your suggestions might be selected for the cover of the journal.

You can now use a single sign-on for all your accounts, view the status of all your manuscript submissions and reviews, access usage statistics for your published articles and download a record of your refereeing activity for the Nature journals.

To assist our authors in disseminating their research to the broader community, our SharedIt initiative provides you with a unique shareable link that will allow anyone (with or without a subscription) to read the published article. Recipients of the link with a subscription will also be able to download and print the PDF.

As soon as your article is published, you will receive an automated email with your shareable link.

With kind regards,

P.S. Click on the following link if you would like to recommend Nature Plants to your librarian
http://www.nature.com/subscriptions/recommend.html#forms

** Visit the Springer Nature Editorial and Publishing website at www.springernature.com/editorial-and-publishing-jobs for more information about our career opportunities. If you have any questions please click here.**

Open Access This Peer Review File is licensed under a Creative Commons Attribution 4.0 International License, which permits use, sharing, adaptation, distribution and reproduction in any medium or format, as long as you give appropriate credit to the original author(s) and the source, provide a link to the Creative Commons license, and indicate if changes were made. In cases where reviewers are anonymous, credit should be given to 'Anonymous Referee' and the source.
The images or other third party material in this Peer Review File are included in the article's Creative Commons license, unless indicated otherwise in a credit line to the material. If material is not included in the article's Creative Commons license and your intended use is not permitted by statutory regulation or exceeds the permitted use, you will need to obtain permission directly from the copyright holder.
To view a copy of this license, visit https://creativecommons.org/licenses/by/4.0/

Dear reviewers and editor,
Thank you for the evaluation of our manuscript and for the constructive input. We have responded to all the comments by either adding new experiments to the manuscript or changing the manuscript text. Please find the responses below; we also attach the manuscript with all the changes highlighted.
On behalf of the authors, Pavel Krupař and Matyáš Fendrych

Reviewers' Comments:

Reviewer #1 (Comments for the Author):

In this manuscript, the authors measured the pH value in the cell walls of root tips using a novel fluorescent indicator. They also investigated the effect of auxin on apoplastic pH in root tips to evaluate the applicability of the classic "acid growth" theory. The findings contribute to our understanding of pH dynamics in the apoplast of various root tip cells and the relationship between apoplastic pH and root growth rate. The results are both novel and interesting. However, there are still some points that need to be addressed or clarified.

Major points:
1. The relationship between apoplastic pH and cell growth rate needs clarification. It appears that root cells in the elongation zone, which experience a higher strain rate, possess a higher cell wall pH. This observation suggests a negative correlation between cell wall pH and cell elongation rate. However, the cell elongation rate is higher in acidic conditions (pH 5.5) compared to neutral conditions (pH 7.0); cell wall acidification induced by FC promotes cell elongation; and IAA-induced cell wall alkalinization, indicating a potential positive correlation between cell wall acidification and cell elongation. These findings seem contradictory, making it difficult to draw a definitive conclusion. In my opinion, it is inappropriate to assert that "root elongation rate is independent of cell wall pH," as cell wall pH does indeed affect root growth.

We agree with this note: the developmental zonation along the root axis seems pH-indepedendent; in other words, pH drop does not seem to be the trigger of differentiation. On the other hand, an artificial manipulation of apoplastic pH will influence growth quickly.
This is the reason that we state in the title that *Root developmental zonation (rather than elongation rate) is independent of cell wall pH.* In the abstract we conclude that "*while on the organ level apoplast acidification promotes root growth, the cellular surface pH and strain rate profiles are largely independent of each other*". We think this is one of the crucial contributions of our paper, because the literature often mixes organ-level growth with cell elongation and assumes that longer cells automatically mean more acidic walls.
To address this comment, we have done our best to make this issue clearer in the abstract and results (lines 24, 65-66, 93-95, 176-179, 257-258).

2. The authors selected a concentration of 100 nM IAA to treat the roots, as this level appeared to inhibit root growth (as shown in Figure S4A). Root cells are more sensitive than those in other organs, and auxin promotes growth at low concentrations while inhibiting it at high concentrations. Therefore, it is important to investigate the effects of lower concentrations of IAA.

As the reviewer suggested, we tested the effects of lower auxin concentrations (0.1, 1, 1 and 10 nM IAA) on root growth and cell wall pH. The 1 and 10 nM IAA concentrations triggered a mild growth inhibition, and an insignificant trend in cell wall alkalinization. We did not observe growth promotion in any of the concentrations tested. The growth promotion by low auxin concentrations might require specific conditions, as was shown by Mulkey et al. (1982, Plant Physiology). We included the new data as Fig.S4 C and D, and introduced it in the results section (line 190-195).

3.The authors observed an asymmetric distribution of cell wall pH at the tips of main roots and young lateral roots influenced by gravity; however, the results from the two experiments seemed inconsistent. The reason for such a discrepancy should be addressed in detail.

Indeed - we could detect clear cell wall pH gradient in gravistimulated lateral roots, but not in the primary roots.
The reason for this discrepancy could be biological or technical. The technical reason could be that the UBI promoter used in the WALLΦ line is particularly strong in the root cap of the primary root, potentially masking the gradient.
To test this possibility, we generated new lines that express WALLΦ from a weaker, tissue specific pCNGC14 promoter that is active in outer tissues of the root. Nevertheless, the new lines with weaker signal in outer tissues also did not reveal any measurable gradient in the cell wall pH in gravistimulated primary roots (Figure S4 G,H,I).
We therefore argue that the reason for the discrepancy between primary and lateral roots is the presence of cuticle on the lateral roots (Berhin et al 2019) that leads to a more pronounced pH changes in the apoplast of the lateral roots. Considering the findings that only 100nM IAA treatment triggered a significant change in cell wall pH, while the lower concentration had no detectable effect, we propose that the cell wall pH is rather stable in comparison to the changes of root surface pH. It is therefore possible that the ion fluxes through the cell wall might not affect the cell wall pH itself, and rather reflect the changes in turgor pressure in the epidermal cell walls of the root.
We included the new results and their discussion on the lines 213-222.

Minor points:
1. In Fig. 3E, it appears that the addition of 0 nM IAA leads to a decrease in pH. What could explain this observation?

The apparent decrease of the sensor ratio over time is likely caused by technical issues: In Fig. 3E, individual segments of cell walls were measured over time, and the shifts in ratios can be caused by shifts of the cell walls in the z dimension as well as by bleaching of the fluorophores. We added the individual curves into the graph in 3E to highlight the variability of the measurements; the crucial message of the graph is that upon auxin treatment, the mock and control samples start to clearly differ.

2. In the legend of Fig. 2, line 265, the authors reference “In G and G.” However, there is no statistical data presented in Fig. 2G.
Thank you for spotting the mistake, it should have been in H and I. We corrected the mistake.

3. In the Materials and Methods section, lines 292-314, the references are cited in a format that differs from that used in the main text.

Thank you for careful proofreading, we unified the citations in this section.

4. In lines 326-327, the notation for “H2O” should have the “2” in subscript.

Thank you for spotting the mistake. It was corrected.

5. In the legend for Fig. S4, line 45, it mentions “Fig. 4AB” and “Fig. 4CD.” However, I cannot find Fig. 4; should it be “Fig. S4”? If so, note that there is no figure D included in Fig. S4.

Thank you for spotting this mistake. We meant “Fig. 3AB and Fig.3CD” and it is now corrected in the text.

6. In Fig. S4A, it is difficult to understand how the root growth rates of “cell wall” and “surface” are measured. Could you clarify this data?

The root growth measurements refer to experiments measuring root cell wall pH and surface pH in Fig.3 AB and CD, respectively. Together with the reaction of cell wall and surface pH to 100 nM auxin, we also measured auxin-induced root growth inhibition. We modified the description of the figure S4 to clarify the issue, and described the method in lines 408-409.

Reviewer #2 (Comments for the Author):

Using WALLΦ, a novel genetically encoded sensor the authors show that although apoplastic acidification promotes root growth at the organ level, local cell wall pH does not correlate with cellular expansion rates. They suggest that instead of directly instructing local growth, cell wall acidity acts as a global permissive factor that enables rapid adjustment of growth rate and direction. The relationship between auxin, cell wall pH and growth rate is central for understanding plant growth and morphogenesis and has received increasing attention in the

past years. However, results using different methods to measure cell surface and apoplastic pH so far have not been conclusive and the field clearly is need of better tools. The chosen approach seems highly promising but several points need to be addressed before this ms becomes ready to go:

- Please describe how strain rate relates to growth velocity. This might be clear to the expert but not to the average reader/reviewer.

We improved the explanation of the logic and terminology of using strain rate measurement in the new version of the paper, see lines 65-66. Strain rate was calculated as derivation or the slope of longitudinal velocity. We have chosen to use strain rate because it is more biologically informative, as it reflects spatial distribution of growth, rather than nominal displacement of the root tip in case of growth velocity.

- What is the evidence that WallPhi actually binds cellulose? Based on the images provided the signal is much more patchy than the CF signal.
- What makes it different from apoPhusion?

We show that WALLΦ binds to the cell wall using a biochemical approach; the sensor was present in the cell wall fraction as determined by the Feiz et al. 20116 method, which ensures that the vast majority of intracellular proteins was washed out and only proteins bound to the cell components remained in isolated fraction, as we point out in lines 121-125.
To support these results using an independent method, we added STED superresolution imaging of lines expressing WALLΦ or the published apopHusion sensor. We were able to observe a pattern clearly resembling cellulose microfibrils in WALLΦ (Fig. 1E), while we never detected such structures in the apopHusion line (Fig. S2C), which uses a similar ratiometric system of two fluorescent proteins, but lacks the carbohydrate-binding module. We also added supplemental videos showing the long term (12 hours) imaging of WALLΦ and apopHusion which clearly demonstrate that the targeting of the sensor to the wall improves the visualization of the apoplastic space. We added the description of the new data to lines 109-112.

- Could the difference between lateral and horizontal wall be an imaging artifact based on different birefringence/material anisotropy?
This is an interesting possibility that we had not considered. We believe this is however not the case for the following reasons:
i) The difference of laterals vs transversal cell walls depends on auxin influx - in the *aux1* mutant this difference is significantly lower (Fig.3K, L). If the effect was optical, aux1 mutation should not influence the sensor readout.
ii) The transversal cell walls react to manipulations of cell wall pH, on Fig. 2F, the increase of transversal cell wall pH is clearly visible
iii) PM-ApoAcidin4 uses a different mechanism of apoplast targeting (is attached to the outer leaflet of PM), yet it shows the same pattern of more acidic transversal apoplast in the elongation zone (Fig. S3B).

- Call me old-fashioned but alkaline is reserved to pH>7, all measurements here show that the cell wall is more or less acidic.

We agree that the highest pH value estimated in the root transition zone (5.5) is still lower than pH 7. We changed the terminology throughout the text as the reviewer suggested. However, we still use the term ‘alkalinization’ for the process of pH increase in the apoplast or cell wall.

Dear Editor,

Please find our final submission attached. We fulfilled all the requirements listed in the Author Checklist.
Below, please find the Brief Summary of the main findings, as requested in the Author Checklist.

Thank you

On behalf of the authors,

Pavel Krupař and Matyáš Fendrych

Brief Summary:

The authors developed a cell wall–anchored pH sensor and found that the cell elongation profile does not correlate with cell wall acidification in Arabidopsis roots. At the same time, cell wall pH changes allow for quick adaptations of growth rate and direction.
